# Male-biased migration from East Africa introduced pastoralism into southern Africa

**DOI:** 10.1186/s12915-021-01193-z

**Published:** 2021-12-07

**Authors:** Mário Vicente, Imke Lankheet, Thembi Russell, Nina Hollfelder, Vinet Coetzee, Himla Soodyall, Michael De Jongh, Carina M. Schlebusch

**Affiliations:** 1 Human Evolution, Department of Organismal Biology, Uppsala University, Uppsala, Sweden; 2 Department of Archaeology and Classical Studies, Stockholm University, Stockholm, Sweden; 3 Centre for Palaeogenetics, Stockholm, Sweden; 4 School of Geography, Archaeology and Environmental Studies, University of the Witwatersrand, Johannesburg, South Africa; 5 Department of Biochemistry, Genetics and Microbiology, University of Pretoria, Pretoria, South Africa; 6 Division of Human Genetics, School of Pathology, Faculty of Health Sciences, University of the Witwatersrand, Johannesburg, South Africa; 7 Academy of Science of South Africa, Pretoria, South Africa; 8 Department of Anthropology and Archaeology, University of South Africa, Pretoria, South Africa; 9 Palaeo-Research Institute, University of Johannesburg, Johannesburg, South Africa; 10 SciLife Lab, Uppsala, Sweden

**Keywords:** Pastoralism, Southern Africa, Hessequa, Khoekhoe, Khoe-San

## Abstract

**Background:**

Hunter-gatherer lifestyles dominated the southern African landscape up to ~ 2000 years ago, when herding and farming groups started to arrive in the area. First, herding and livestock, likely of East African origin, appeared in southern Africa, preceding the arrival of the large-scale Bantu-speaking agro-pastoralist expansion that introduced West African-related genetic ancestry into the area. Present-day Khoekhoe-speaking Namaqua (or Nama in short) pastoralists show high proportions of East African admixture, linking the East African ancestry with Khoekhoe herders. Most other historical Khoekhoe populations have, however, disappeared over the last few centuries and their contribution to the genetic structure of present-day populations is not well understood. In our study, we analyzed genome-wide autosomal and full mitochondrial data from a population who trace their ancestry to the Khoekhoe-speaking Hessequa herders from the southern Cape region of what is now South Africa.

**Results:**

We generated genome-wide data from 162 individuals and mitochondrial DNA data of a subset of 87 individuals, sampled in the Western Cape Province, South Africa, where the Hessequa population once lived. Using available comparative data from Khoe-speaking and related groups, we aligned genetic date estimates and admixture proportions to the archaeological proposed dates and routes for the arrival of the East African pastoralists in southern Africa. We identified several Afro-Asiatic-speaking pastoralist groups from Ethiopia and Tanzania who share high affinities with the East African ancestry present in southern Africa. We also found that the East African pastoralist expansion was heavily male-biased, akin to a pastoralist migration previously observed on the genetic level in ancient Europe, by which Pontic-Caspian Steppe pastoralist groups represented by the Yamnaya culture spread across the Eurasian continent during the late Neolithic/Bronze Age.

**Conclusion:**

We propose that pastoralism in southern Africa arrived through male-biased migration of an East African Afro-Asiatic-related group(s) who introduced new subsistence and livestock practices to local southern African hunter-gatherers. Our results add to the understanding of historical human migration and mobility in Africa, connected to the spread of food-producing and livestock practices.

**Supplementary Information:**

The online version contains supplementary material available at 10.1186/s12915-021-01193-z.

## Background

Hunting and gathering was the only lifeway practiced in southern Africa until approximately 2000 years ago. Previous studies have suggested that at that time, a herding group of East African origin introduced herding practices and livestock into southern Africa and admixed with local hunter-gatherer groups to form what became known as the Khoekhoe populations [1–6]. This group of East African origin was an already admixed group with both East African and Eurasian genetic components (69% East African and 31% Eurasian ancestry), comparable to the present-day Amhara and Oromo groups from Ethiopia [2, 6]. The East African migration into southern Africa was shortly followed by an independent and separate agro-pastoral migration into the region, the Bantu expansion, which introduced a West African genetic component into southern Africa [1, 7, 8]. Bantu speakers across sub-Saharan Africa have a clearly distinguishable West African genetic ancestry, irrespective of their present-day location [9–12]. While the Bantu expansion, and subsequent European-driven colonialism and slave trade, have culturally replaced many traditional hunter-gatherer and pastoralist practices, these traditions are still present in scattered groups across southern Africa. Present-day southern African hunter-gatherers (San) and herders (Khoekhoe) are collectively referred to as Khoe-San. Khoe-San people speak Khoisan languages, a group of languages that rely heavily on “click” sounds. Southern Africa hosts three out of five major Khoisan language families (Additional File 2: Table S1), namely: Kx’a (formerly called Northern Khoisan), Tuu (formerly Southern Khoisan) and Khoe-Kwadi (formerly Central Khoisan). These three language families show no linguistic relatedness to each other [13].

In contrast to Kx’a and Tuu that are spoken exclusively by hunter-gatherers, Khoe-Kwadi was historically spoken by hunter-gatherers and pastoralists. The Khoe-Kwadi language family can be structured in three sub-branches: Kalahari Khoe, Khoekhoe and Kwadi (extinct) [14] (Additional File 2: Table S1). The Kalahari Khoe speakers were historically hunter-gatherers, although some of these groups, for example the |Gui and G||ana, have a record of keeping livestock while retaining a hunter-gatherer subsistence base [15–18]. Khoekhoe and Kwadi speakers, on the other hand, are known to have been pastoralists [14, 19]. The Kwadi language from Angola disappeared over the last few decades, and Khoekhoe speakers today are limited to the Nama and Hai||om from Namibia. However, Khoekhoe languages used to have a wider distribution in the sub-continent. !Ora, Eini and various Cape Khoekhoe languages belonging to the Khoekhoe family were once spoken in the southernmost part of the African continent (Additional File 1: Figure S1) [20]. Historical records from the European colonial period in the Cape report the presence of herders, presumed to be Khoe-language speakers, along the west, south and southeast coasts of southern Africa with large flocks of domesticated animals [21–24].

Khoekhoe pastoralists have been linked to the introduction of livestock to southern Africa for many decades but this has been overwhelmingly based on linguistic evidence [14, 19, 25]. Early archaeological studies suggested that hunter-gatherer groups acquired their initial livestock through interaction with Bantu speakers in an area north of South Africa (suggested transfer areas were southeastern Angola, southwestern Zambia, Zimbabwe, or northern Botswana), which was followed by their spread southwards among Khoe-San populations [26–29]. However, more recently, studies have rather supported that pastoralists from East Africa migrated to southern Africa and interacted with autochthonous hunter-gatherers, introducing herding skills and East African-origin domesticated animals into the area [14, 30]. Radiocarbon dating of livestock remains pinpoint the earliest sheep and cattle in southern Africa to around 2000 years ago [31–35]. Linguistically, the link between pastoralism in East and southern Africa is supported by the shared relatedness of Khoe-Kwadi languages with Sandawe (an East African Khoisan language), particularly the relationship between Sandawe with Kwadi and Khoekhoe [14, 20]. These findings also received support from genetic studies [1, 2, 6, 11, 36].

Ancient DNA studies on human remains demonstrate that all extant Khoe-San groups have admixture with a mixed group containing East African and Eurasian ancestry [2, 4]. This East African-Eurasian component is present in the highest fractions among Khoe-speaking groups [1, 2, 4, 5, 37, 38]. The East African genetic contribution also introduced adaptive genetic variants into southern Africa. Khoekhoe speakers show relatively high frequencies of the “East African” lactase persistence (LP) polymorphism (*C-14010* or *rs145946881*) allowing the digestion of milk in adulthood [5, 39]. In contrast, this LP variant is at low frequencies or absent in the various San hunter-gatherer groups. There are several region-specific LP variants that were selected in pastoralist societies where diets rely heavily on dairy products [40–43].

The present-day Western and Eastern Cape provinces in South Africa were once home to many, now extinct, Khoekhoe languages, linguistically grouped as Cape Khoe (Additional File 1: Figure S1). Based on the limited records, it is known that Cape Khoe people shared common Khoekhoe socio-cultural practices despite some variation between groups [44–46]. Among those Cape Khoe speakers, the Hessequa (meaning of name: Hesse—trees/woods (an adjective suffix), qua—people of) were a group that lived in the eastern part of the Western Cape province region (Additional File 1: Figure S1). Initial documentation by European travelers in the seventeenth century reported the Hessequa to be Khoekhoe people with large numbers of cattle and sheep [47]. However, during colonial times, the Hessequa, like all Cape Khoe speakers, were severely affected by infectious diseases introduced by European colonists, causing several major smallpox epidemics and influenza outbreaks [48, 49]. These epidemics, coupled with colonial warfare and unfair trading practices, affected the survival of the Cape Khoe groups who were assimilated by a colonial-imposed segregated society. They lost their languages and the Khoekhoe cultural identity, to become part of the new mixed ancestry “Coloured” population [19, 50]. In this article, we use the term Coloured following the current-day continued use of the term as self-identification (“Coloureds”. Retrieved, from [51]).

In this study, we generated genome-wide data from 162 individuals sampled in the Western Cape Province, South Africa, where the Hessequa population once lived. Surnames, landownership records and oral histories connect the study participants to the original Hessequa population of the region [47]. Together with previously published genetic data from Khoe-speaking and other comparative groups, we provide new insights regarding the ancestry of the Hessequa people and the history of the introduction of pastoralism to southern Africa. We also sequenced the full mitochondrial genome (mtDNA) of 87 Hessequa descendants (randomly selected) and evaluate sex-specific demographic patterns using autosomal, X and Y chromosome variant sites together with mtDNA data.

## Results

**Fig. 1:**
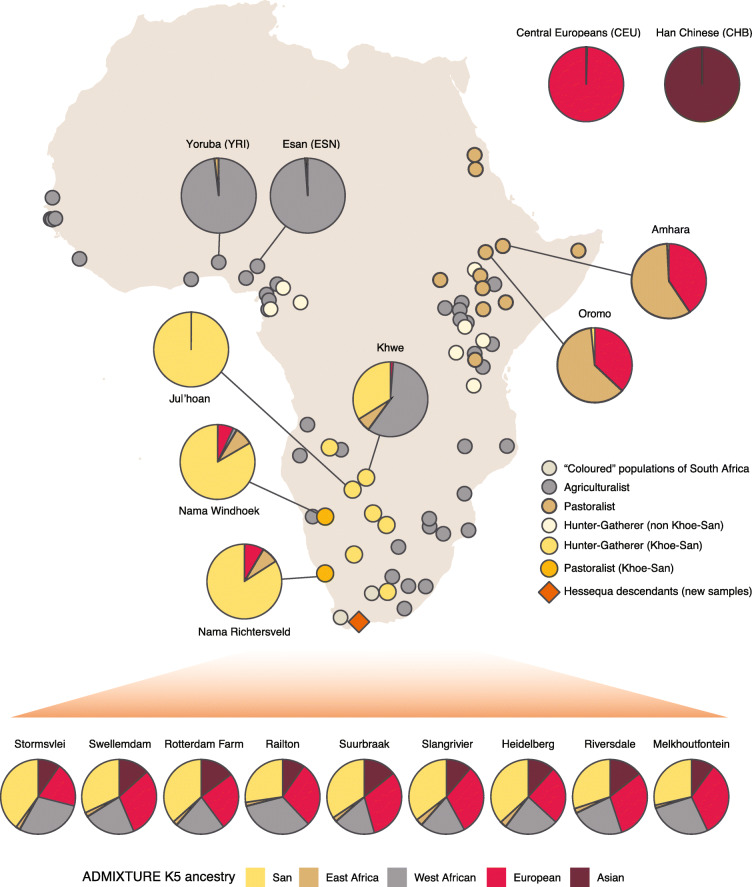
Geographic location and genetic ancestry assignment of the samples used in this study. Pie charts are averaged cluster assignments at *K* = 5 (ADMIXTURE analysis) for relevant populations (complete cluster analysis is available in Figure S2). The Hessequa descendants’ ancestry proportions were calculated for the nine sampling sites separately. Full population description and origin of datasets are summarized in Additional File 2: Table S8

We started by investigating the genetic structure of Hessequa descendants in comparison to African (and worldwide) datasets (Fig. 1). We estimated unsupervised ancestry fractions from an assumed number of clusters (*K* = 2 to *K* = 10, Additional File 1: Figure S2, S3). At *K* = 5, the ancestry components reflect the five major genetic ancestries present in the dataset: Southern African San (yellow), East African (brown), West African (gray), European (red) and Asian (dark-red). We report ADMIXTURE results for *K* = 5 in detail since it is the *K* with the lowest cross-validation error (Additional File 1: Figure S4), without southern Africa Khoe-San groups showing signals of Rainforest hunter-gatherer related ancestry, which is likely due to shared ancestry among groups. We observe that Hessequa descendants from the nine sampling sites show signatures of multiple distinct ancestries with component contributions similar across all sampling sites. The Hessequa descendants at *K* = 5 show their autochthonous southern Africa San ancestry ranging from 27.3 to 40%, while the East African-associated ancestry showed proportions ranging from 1.3 to 3%. The other ancestry fractions were 17.9–33% West African-, 19.3–32.9% European- and 9.6–14.7% Southeast Asian-related ancestries. Similar ancestry composition patterns are also visible in Coloured populations from two other regions of South Africa (Colesberg and Wellington), with a San ancestry of 37.7% and 19.1%, respectively [1, 52]. However, only the Coloured population from Wellington show an East African-related ancestry fraction (1.1%), which is not observed in the Coloured populations from Colesberg. To formally test if the genetic affinities of the Hessequa descendants were the result of admixture, we performed an admixture graph [53] under a proposed demographic model (Additional File 1: Figure S5). A model that describes the Hessequa descendants as an admixed group between the ancestors of southern Africa Stone Age hunter-gatherers (San) mixed with East African pastoralists, a West Africa-related ancestry group and a European and Asian ancestry group, could not be rejected (*Z*-score = 2.424).

**Fig. 2:**
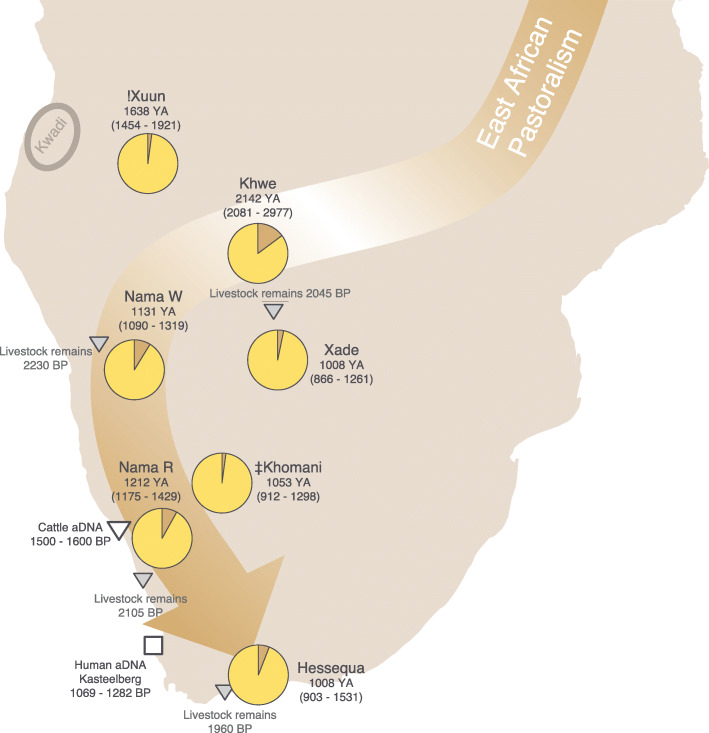
Geographic representation of the pastoralist arrival into southern Africa. Pie charts represent the proportions of East African and San ancestry exclusively (Additional File 2: Table S2), with admixture dating for the tested population. The Hessequa descendants’ ancestry fractions and date estimations are based on the selected 58 Hessequa descendants. Ancestry fractions of the complete sample set are presented in Additional File 2: Table S2. White square: Geographic location of a human individual buried in pastoral context from whom aDNA was obtained. White triangle: Approximate geographic location of early cattle remains with ancient DNA yield and directly radiocarbon dated. Gray triangles: Geographic coordinates of the earliest findings of livestock remains (Approx. date included based on stratigraphy)

The East African ancestry is visible in several Khoe-San groups from southern Africa (Additional File 1: Figure S2). It is present in Khoe-Kwadi speakers (Nama Windhoek, Nama Richtersveld, Xade, Khwe), together with ǂKhomani (Tuu speakers) and !Xuun (K’xa speakers). For the Khoekhoe-speaking Nama, sampled in the Richtersveld (South Africa) and Windhoek (Namibia), the East African-related ancestry is the highest among all southern Africa Khoe-San groups with 7.4 and 8%, respectively. In the Kalahari Khoe, the same ancestry shows smaller fractions with 6.1% in the Khwe and 3% in the Khoe-San from Xade. Interestingly, we do not see the East African-related ancestry in the gene pool of the San from Khutse (previously labelled as |Gui and G||ana) but we detect small East African proportions in the Tuu-speaking ǂKhomani (1.9%) and the K’xa-speaking !Xuun (1.6%). If the East African ancestry is assumed to have been introduced into southern Africa by a single event, prior to subsequent arrival of other genetic components, the contribution of the East African fraction in relation to the San ancestry can possibly provide insights about the diffusion of the East African-related component in southern Africa (Additional File 2: Table S2). We observe that Khwe have the highest proportion of East Africa-related ancestry in relation to their original San-related ancestry with 15% followed by the Nama populations from Windhoek and Richtersveld with 9 and 8%, respectively. Hessequa descendants have an East African- to San-associated fractions with a ratio of 6% average, with ratios ranging from 8.9% in Slangrivier to 4.8% in Melkhoutfontein (Fig. 2, Additional File 2: Table S2). We also observe that the 156 unrelated Hessequa descendants carry the East African-associated lactase persistence mutation (C-14010) at frequency of 12.9% (Additional File 2: Table S3), followed by European-associated mutation at 12.0% (A-22018, in strong LD with T-13910—not represented on the H3Africa SNP array).

To date the times when the non-San ancestries were incorporated in the Hessequa descendant population, we determined the different ancestry segments across the genome and used the linkage disequilibrium (LD) information to infer time estimates [54]. We initially estimated the different local ancestries without providing any prior information of the potential parental sources under a 5-way admixture model (Additional File 1: Figure S6). We observe that the East African ancestry segments have higher affinity with Borana, Iraqw, Oromo, Datog, Amhara, Rendille and Burji populations. All of these groups are (or were historically) Afro-Asiatic speaking pastoralist populations from East Africa, with the exception of the Datog pastoralists who speak a Nilo-Saharan language. Interestingly, similar genetic affinities are observed among the East African segments of other Khoe-San groups suggesting a common origin for East Africa-related ancestry in southern Africa (Additional File 1: Figure S7-S12). The dating estimation using pairwise co-ancestry curves between San and East Africans in the Hessequa descendant population, however, show spurious results (Additional File 1: Figure S13). In the case of an admixture event, a specific allele of source A is more likely to find an allele of source B as physical distance between the alleles increases. However, the co-ancestry curve in the Hessequa descendants for the San and East African ancestries do not show this pattern. These two ancestries are more likely to be found within 10 Mb of each other but not further apart. Also, the San and East African ancestries are not completely intertwined either, as expected in a null model of admixture (would result in the classic LD decay pattern). To minimize the complexity of the demographic scenario, we used a 3-way admixture model instead and use the Ju|’hoan, Amhara and Gujarati from India (GIH) as parental sources for the San, East Africa and a combined Eurasian proxy (since the European and Southeast Asian ancestries admixed with the Hessequa at similar times, see further down), and date the East African ancestry to 951 years ago (CI 903–1531, Additional File 1: Figure S14, Additional File 2: Table S4), assuming 30 years per generation time. When the Hessequa descendant groups are analyzed individually, we date the arrival of East African ancestry averaging between 567 (CI 313–948) and 1287 years ago (CI 1036–1938) (Additional File 2: Table S4). The West African, Southeast Asian and European ancestries seem to have entered the Hessequa descendant gene pool much more recently, during the colonial period of South Africa, with dates of 234 (CI 207–291), 213 (CI 173–250) and 192 (CI 169–228) years ago, respectively. The West African-related segments have highest affinities with southeast Bantu-speaking populations from South Africa (Venda and Tsonga groups) and Mozambique (Makhuwa, Ndau, Nyanja and Bitonga groups) (Additional File 1: Figure S6).

We further estimated the arrival of the East African ancestry across southern Africa by dating admixture times in other Khoe-San groups (Fig. 2, Additional File 2: Table S4). The earliest time estimate for the East African-related ancestry in southern Africa was observed in the Kalahari Khoe-speaking Khwe of southern Angola, dating to 2142 years ago (CI 2081–2977, Additional File 1: Figure S15). Although their main genetic ancestry is a West African-origin Bantu speaker component, the admixture between their San- and their East African-related segments predates the admixture with Bantu speakers (with West African segments admixing with the San and East African ancestries at similar times—576 years ago (CI 508–645) and 597 years ago (CI 506–804). Subsequently, the East African ancestry seems to follow the western coast and admixture time estimates become more recent the more southward the location of populations. The same pattern was observed moving from the coast to inland populations although confidence intervals partially overlap. For instance, the K’xa-speaking !Xuun, living in southern Angola date their 1.6% East African ancestry to 1638 years ago (CI 1454–1921, Additional File 1: Figure S16). The Khoekhoe-speaking Nama from the Richtersveld and Windhoek date their East African ancestry to 1212 (CI 1175–1429) and 1131 (CI 1090–1319) years ago, respectively (Additional File 1: Figure S17, S18). The Khoe-San from Xade in the Central Kalahari Game Reserve date the arrival of East African component to about a millennium (1008 years ago, CI 866–1261) ago (Additional File 1: Figure S19). Finally, the Tuu-speaking ǂKhomani show low levels of an East African ancestry dated to 1053 years ago (CI 912–1298, Additional File 1: Figure S20). The decreasing admixture dates from North to South therefore possibly reflect the migration route, however many of the date estimates had overlapping confidence intervals (Fig. 2, Additional File 2: Table S4). Only the !Xun and Khwe in the north had significantly earlier dates, which might indicate a pause after the initial admixture and a subsequent rapid spread to the south.

**Fig. 3:**
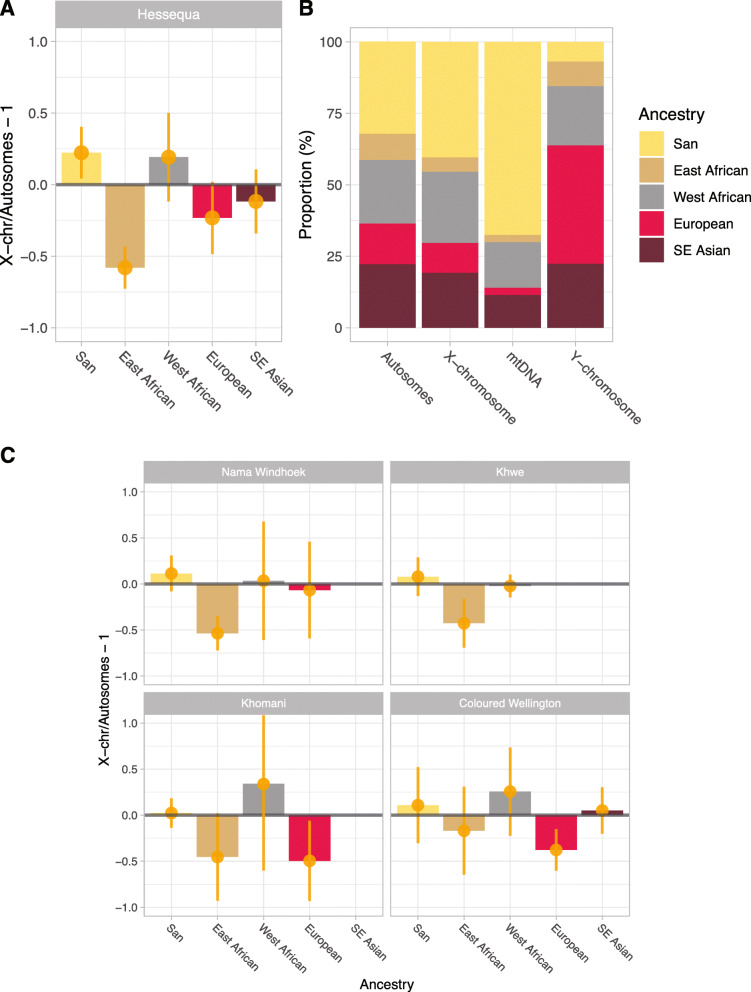
Sex-biased admixture estimates among southern Africa Khoe-San and their descendent groups. **A** X chromosome to autosomal ratio for the Hessequa descendants based on the average ancestry proportion. Autosomal data is represented by the first 180 cM of chr 1–7, 10 and 12. Error bars represent two standard deviations based on 100 random sampling bootstraps. **B** Averaged ancestry proportion of supervised admixture for autosomes and X chromosome. Ancestries of mtDNA and Y chromosome haplogroups were assigned according to the haplogroup geographic prevalence. **C** X chromosome to autosomal ratio for the Khwe, Nama from Windhoek, ǂKhomani and Coloured population of Wellington

We next explored whether the East Africa admixture in the Khoe-San was a result of sex-biased admixture. For datasets where the X chromosome data were available, we estimated the X-to-autosomal (X/A) ratio from the admixture fractions in Khoe-San populations with an average East Africa ancestry in autosomes and X chromosome higher than 2% (based on a 5-way supervised admixture analysis): the Hessequa descendants, Nama from Windhoek, Khwe, ǂKhomani and Coloured population of Wellington. Most populations have a X/A ratio above 1 for San ancestry, indicating a higher female-to-male ratio for the San ancestry (Fig. 3A, Additional File 2: Table S5). On the other hand, the East African ancestry seems to be male-biased; with an X/A ratio of 0.42 ± 0.07 SD in the Hessequa descendants, 0.46 ± 0.09 SD in the Nama, 0.57 ± 0.13SD in the Khwe, 0.54 ± 0.23 SD in the ǂKhomani and 0.83 ± 0.23 SD in the Coloured population of Wellington. Similarly, we also see male-driven admixture for the European ancestry, as expected [55], with X/A averages ranging between 0.50 and 0.93.

Although the X chromosome contains many more independently segregating loci (due to recombination of the X chromosome in females), the single uniparental loci, mtDNA and Y chromosomes, should, to some extent, reflect the same patterns observed in the X/A ratio. Since haplotype inference from SNP-chip data is unreliable [56], we generated full mitochondrial genomes for 87 Hessequa descendants (7 to 11 individuals from each sample site, picked randomly) and assigned an ancestry for each haplotype according to population groups in which these haplogroups were highly prevalent in previously published studies (Additional File 2: Table S6) [57–59]. A total of 58 out of 87 Hessequa descendants carried mtDNA with the haplogroup L0d (Additional File 2: Table S6). L0d lineages are almost exclusively found in Khoe-San populations [60–62], indicating that 66.7% of the Hessequa descendants mtDNA gene pool is autochthonous to the region (Fig. 3B). L0d1b is the most prevalent lineage of L0d haplogroup in the Hessequa descendants (62.1% of the L0d lineages) and occurs commonly across all Khoe-San populations and Coloured groups from the Cape [60–62]. The second most observed L0d lineage is L0d2a (31.0% of the L0d lineages). This sub-haplogroup was noted to have an almost exclusive presence among southern Khoe-San (Nama, ǂKhomani and Karretjie), with attributed evidence for a recent and fast spread [62]. Typically East African-associated lineages were present in only two Hessequa descendants (haplogroups L4b and L5a), while West African haplogroups linked to the Bantu expansion were detected in 14 (16.3%) individuals (L0a, L1b, L2a, L3d and L3e). We also observe 11 (11.6%) Asian mtDNA haplogroups (B, E, M, U7a), with haplogroup M2a as the most prevalent Asian haplogroup. Finally, only two individuals (2.3%) had European mtDNA haplogroups, one individual with haplogroup H and another with haplogroup J1c. One individual carried the U2a1a mtDNA lineage, a relatively rare haplogroup that has its highest distribution in central Eurasia; therefore, we could not classify it as Asian or European origin.

To compare with our mtDNA results, we use the Y chromosome markers present in the H3Africa SNP array and classified their Y chromosome haplogroups using SNAPPY [63] (Additional File 2: Table S7). We observe that only four Hessequa descendants hold San-related A1b1 lineages, while five individuals carried the E1b1b (E-M35) Y chromosome haplogroup, associated with East African pastoralists [64]. Our results indicate that the Hessequa descendant Y chromosome gene pool is composed of 6.9% San, 8.6% East African, 20.7% West African, 41.4% European and 22.4% Asian ancestry. Previous uniparental markers studies on SAC populations have shown haplogroup frequencies in line with the ones observed in the Hessequa descendants [52, 57, 62, 65]. For example, a study on a Western Cape Coloured group [57] has reported the San-associated mtDNA haplogroup L0d frequency of 60.0% [57], correlating with the high frequencies in the Hessequa descendants (66.7%). In both studies the San-associated Y chromosome haplogroup A1b was observed in much lower frequencies compared to San-associated mtDNA haplogroups (Hessequa descendants: 6.9%; Western Cape Coloured [57], 5.3%).

## Discussion

The Southern African San represent one of the two first branches of the human population tree, with all other extant humans belonging to the other branch [1–3, 11, 66–68]. San ancestors were likely to be the only inhabitants of southern Africa during most of prehistory [2, 6], with geographic proximity reflecting their genetic structure under an isolation-by-distance model [69]. Pastoralism in southern Africa is linked to the arrival of East African group(s) around ~ 2000 years ago, preceding the West African ancestry Bantu-speaking farmers arrival in the region [34, 70–72]. Among Khoe-San groups, the East Africa pastoralist migration impacted present-day Khoe-Kwadi speaker genomes in particular, but not exclusively (Fig. 1, Additional File 1: Figure S2). This East African admixture in Khoe-San groups has been reported previously [1, 4] and later confirmed by aDNA analyses of ~ 2000 years old Late Stone Age individuals from present-day South Africa [2, 6].

Assessing, on the molecular level, when East African pastoralists arrived into southern Africa has been challenging due to the impact of additional gene flow from other immigrant populations. The Bantu expansion reached the area approximately 500 years after the East African pastoralist arrival and influenced the genetic variation (and geographic distribution) of the Khoe-San groups. Additional inter- and intra-continental genetic ancestries were introduced into the Khoe-San gene pool during the colonial period (1600s onward). This complex history hinders inferences about preceding (and perhaps more subtle) demographic events. Therefore, we applied an ancestry-specific haplotype-based approach to obtain time estimates for East African admixture into the autochthone Southern African gene pool. Khwe speakers had the oldest East African admixture date in our dataset, i.e. 2142 years ago (CI 2081–2977) (Fig. 2, Additional File 2: Table S4). The date and geographic location of the Khwe fit with archaeological evidence for the arrival of pastoralism into the area (Fig. 2, Additional File 2: Table S4) [34, 72]. Although both archaeological and genetic data points are few, it seems that East African admixture dates follow the same route as the appearance of livestock remains across southern Africa: southwards along the Atlantic coast and then moving inland [34, 72].

We note, however, that our inferred admixture dates do not correspond to the earliest archaeological findings in the regions. For instance, the East African admixture event in the Hessequa descendants dates to 1008 years ago (903–1531) but livestock remains found at the Blombos site in the Western Cape started to appear ~ 2000 years ago in the archaeological record [73]. Similarly, we observe a time difference between the earliest presence of sheep and cattle in the area where Nama reside (~ 2175 years ago) [35], and the East African admixture date in the Nama, 1212 years ago (1175–1429). This time discrepancy could be explained by the diffusion of livestock among hunter-gatherer groups ahead of the movement of people [18, 74, 75]. While there is the possibility that the some of the archaeological evidence for the spread and dating of the earliest domestic livestock is unreliable—particularly in the earlier time period when the sample of morphologically identified domestic livestock is small and sparse across South Africa—the earliest dates for cattle and sheep on the west coast of South Africa are both from securely identified and dated samples [33–35]. Both palaeoproteomics [76] and ancient DNA analysis [77–79] of archaeological samples show that sheep and cattle bone have sometimes been misidentified, when they are in fact wild antelope. In South Africa, the earliest secure occurrence of domestic cattle identified by ancient DNA analysis and directly dated is from Namaqualand and dates to about 1500 years BP [33]. This agrees fairly well with the date that this study gives to the East African Ancestry of Khoekhoe-speaking Nama from the Richtersveld at 1212 bp (CI 1175–1429) (Additional File 1: Figure S17, S18). The earliest sheep, identified by palaeoproteomics and directly dated, are from the site of Spoegrivier in the northern Cape of South Africa [35]. The earliest sheep remains identified by ancient DNA are from the sites of Blydefontein, in the northern Cape [80] and Die Kelders 1 on the southern Cape coast. Neither of these have been directly dated. Although date estimates were obtained by association with the stratigraphy, this is unreliable at these sites. The Die Kelders 1 sheep sample is from layer 2 that dates to approximately 1300 years BP [34, 81] and the Blydefontein sheep sample falls anywhere within the last 1000 years BP [82]. More research is needed on the archaeological samples, as well as other potential archaeological markers for novel groups on the landscape, to resolve these issues. A third possible explanation for the discrepancy between inferred admixture dates in this study and the earliest archaeological findings is that the genetically inferred admixture time estimates are average dates from a continuous admixture pulse that might have stretched over a certain timespan. This hypothesis is supported by a significant enrichment of livestock remains in the area that the Hessequa once lived, in a period between 1500 and 1000 years ago [34].

It is worth mentioning that Nama speakers lived exclusively in the present-day Northern Cape province region of South Africa until recently [19]. The date estimates from the Nama living near Windhoek (1131 years ago (1090–1319)) should therefore be similar to the Nama from the Richtersveld (1212 years ago (1175–1429)). Our time estimates for East African admixture into the Nama overlaps with the radiocarbon dates of human remains found at Kasteelberg that yielded aDNA results [6]. The Kasteelberg individual was buried in an archaeological context associated with pastoralism and was carbon dated to have lived around 1200 years ago [6]. The Kasteelberg individual had 40.3 to 54% East African genetic ancestry [6], a substantially higher fraction than modern-day Khoekhoe Nama groups (7.4 and 8% in this study). Additionally, an individual that died ~ 200 years ago in the Vaalkrans Shelter in the Cape south coast region had an intermediate East African ancestry fraction (15 to 32%), thus falling between the Kasteelberg individual and contemporary Khoekhoe pastoralists [83]. Although based on only a few data points and given that ancestry fractions were calculated with different methodologies, it seems likely that the East African ancestry fraction has become diluted over time, possibly due to subsequent gene flow between San hunter-gatherers and Khoekhoe pastoralist groups.

Previous studies suggested that the East African component from modern Khoe-San individuals show high affinities with present-day Afro-Asiatic speakers from Ethiopia, such as the Amhara [2]. Based on our ancestry-specific analysis, we confirm that the East African haplotypes carried by the Khoe-San are found in several Afro-Asiatic pastoralist groups from present-day Ethiopia and Tanzania (Additional File 1: Figure S6-S12). This result is also supported by our admixture model testing in the Hessequa descendants where we used ancient individuals from pastoral Neolithic contexts in Ethiopia and Tanzania [84] (Additional File 1: Figure S5). Our genetic results hint at a possible link between southern African pastoralism and the migration of East African Afro-Asiatic speakers. This finding appears to be in conflict with the linguistic relationship that was inferred between Sandawe (an East African Khoisan language) and the Khoe-Kwadi languages [14]. The Sandawe language, however, seems to have been influenced by neighboring Afro-Asiatic Cushitic languages [85]. The linguistic affinity among Sandawe and Cushitic languages [86], together with the possible link between Sandawe and Khoe-Kwadi languages, and finally, the genetic affinity between Khoe-Kwadi and Afro-Asiatic groups, establish a network of interconnectedness linked to the arrival of pastoralism in southern Africa [87].

The East African-Eurasian mixed ancestry in Khoe-San groups has been reported before [2, 4–6, 39], but the information regarding sex-biased patterns has only been assessed using uniparental markers [61, 68, 88, 89]. Although studies on mitochondrial DNA and the non-recombining part of the Y chromosome (NRY) can provide significant insights, these two markers are transmitted in their entirety, from parents to offspring and therefore represent single lineages (paternal for Y and maternal for mtDNA). Thus, by studying mtDNA and NRY alone, the genetic information from multiple ancestors is not captured. Studying the ancestry fractions of the X chromosome in relation to the fractions observed in autosomes provide more robust insights regarding sex-biased gene flow. Based on the X chromosome to autosomal ancestries ratio (X/A ratio) observed in this study, we propose that the East African pastoralist migration was strongly male-biased (Fig. 3). In addition, the E1b1b Y chromosome haplogroup has been associated previously with the spread of pastoralism into southern Africa [64]. We observed that five Hessequa descendants carried this Y chromosome haplogroup contrasting with lower levels of San-related haplogroup lineages. On the mitochondrial level, very little evidence of East African gene flow has been detected in modern Khoe-San mtDNA, either in this study or previously reported [60–62]. Therefore, evidence from uniparental markers provides further support for a heavily male-biased East African expansion into Southern Africa. A similar male-driven migration has previously been described on the genetic level in ancient Europe, where Pontic-Caspian Steppe herder groups represented by the Yamnaya culture spread across the Eurasian continent during the late Neolithic/Bronze Age [90]. Male-mediated admixture as a dynamic of the interaction between resident communities and incoming groups seem to be a general pattern across several populations on different continents. This pattern has also been reported previously across Southern Africa [88, 89].

The colonization of southern Africa by Europeans from the 1600s onwards, further impacted the genetic landscape of the indigenous communities of southern Africa, particularly in the area where Cape Khoe speakers once lived. Previous studies on Western Cape Coloured populations have reported similar ancestry fractions to the ones observed on Hessequa descendants [1, 2, 38, 91–93]). We note, however, that most previous studies did not report an East African-related ancestry, possibly due to lack of East African reference populations in their comparative datasets.

The genomes of Hessequa descendants trace 58.1 to 70.1% of their genetic ancestry to groups that immigrated into southern Africa during the colonial period. Apart from European admixture from settlers and mariners, they also received genetic contributions from Bantu-speakers and slaves of Southeast Asian ancestry. The West African-related component in the Hessequa descendants has the closest match to Bantu-speaking groups of Mozambique and northeast South Africa (e.g. Venda, Tsonga, Makhuwa, Ndau) but not with neighboring Xhosa speakers. These results could possibly be explained by the fact that Bantu-speaking groups from Mozambique and northeast South Africa have little to no admixture with local Khoe-San speakers [37, 38, 94, 95]. Consequently, they provide a higher affinity to the ancestral population in MOSAIC analyses than other South African Bantu speakers who contain a significant portion of Khoe-San admixture in their genomes. Our results contrast to findings for the Afrikaner community of South Africa, where the Afrikaner West African-associated ancestry had higher affinities with the Yoruba population from Nigeria [96]. Different sources of slaves and different population interactions across geography and time during the colonial era could explain differences in West African ancestries. Alternatively, Hessequa descendant Bantu-speaking ancestry could also have potentially arrived together with their Southeast Asian component, originating from slaves from Madagascar [97] since Malagasy populations have both Southeast Bantu-speaking and Melanesian ancestries [98, 99]. However, our date estimates show that the West African and Southeast Asian ancestries in the Hessequa descendants admixed during the colonial period, which argues against this hypothesis (Additional File 1: Figure S13). Since slaves also arrived from the eastern African coast [97, 100], we note that additional East Africa ancestry could been introduced during the colonial period in the Hessequa descendants through East African Bantu-speaking slaves (who hold a main West African ancestry with minor East Africa composition). This additional source of East African admixture could explain the atypical co-ancestry curve shape (Additional File 1: Figure S11), which could be a result of multiple admixture events at different time points (for explanation please see Figure S6 in [101].

## Conclusion

Our results support previous findings that East African-Eurasian ancestry arrived in southern Africa around 2000 years ago, possibly introducing pastoralism and livestock into the area. Although the complete history of the introduction of pastoralism to southern Africa remains to be uncovered, we detect that the East African component in southern African genomes is genetically most similar to several pastoral Afro-Asiatic speaking groups from Ethiopia and Tanzania. Furthermore, we also inferred that the East African pastoralist expansion was significantly male-driven. This result is supported by different sections of the genome: autosomes vs. X chromosome ancestry ratios, mtDNA and NRY markers. Although based on very few archaeological and genetic data points, we see some similarities and trends between archaeological proposed routes for the spread of pastoralism across the southern Africa region and genetic admixture dates of the East African component into an autochthonous southern African hunter-gatherer background. Apart from the earliest contact between autochthonous hunter-gatherers and the East African-origin group(s), our haplotype-based admixture time estimates tentatively indicate that this East African ancestry might have spread at a slower pace than livestock remains—support is lent to this hypothesis by the archaeological data and ethnographic studies [18, 75, 102].

Finally, we provided additional insights on how the colonial era impacted the genetic diversity of southern Africa, in particular among the groups that trace part of their ancestries to the Hessequa people of the southern Cape coastal region.

## Methods

### Sampling and genome-wide SNP typing

The Hessequa descendant samples were collected in Western Cape, South Africa, after in-depth field work by an anthropologist (MDJ) on the ethnographic background of the communities in the region. Written informed consent was obtained from all 162 participants included in the study before saliva samples were collected. Sample collection of Coloured, Khoe-San and Khoe-San descendent groups were approved by the University of the Witwatersrand Human Research Ethics board, clearance numbers M980553, with renewals M050902, M090576, M1604104. This specific project was approved by University of the Witwatersrand Human Research Ethics board, clearance number M180655 and the National Ethics review board of Sweden, clearance number Dnr 2021–01448. The biological material was collected in 2 mL Oragene saliva kits (DNA Genotek) and DNA was extracted using the prepIT L2P extraction protocol. The 162 samples were genotyped on H3Africa Consortium SNP panel implemented in Illumina Infinium assay (H3Africa_2017_20021485_A2 BeadChip). The data were generated by the SNP&SEQ Technology Platform in Uppsala, Sweden. The data were analyzed using GenomeStudio v.2011.1 and aligned to the Human Genome built version 37. A total of 2,267,346 genomic markers were obtained.

### Quality filtering and autosomal dataset merging

Data management and quality filtering were carried out using the PLINK v.1.90 software [103]. Of the 162 Hessequa descendants, three individuals were excluded due to reported relatedness, one individual failed to pass 0.05 data missingness threshold. We subsequently filtered to keep only biallelic SNPs with a SNP missingness filter of 0.05. To account for possible genotyping errors, we applied a Hardy-Weinberg equilibrium filter (HWE) of 0.005 per sampling location and only overlapping SNPs were excluded. AT and CG SNPs were also filtered out to prevent strand flipping errors when merging with comparative datasets. Cryptic relatedness was inspected by identity by state (IBS) analysis and two samples were removed (one first-degree and one second-degree related). A total of 2,076,226 autosomal SNPs and 156 unrelated individuals were kept for the study. We merged the newly generated data with 1655 comparative samples from 81 populations (Additional File 2: Table S8) [6, 69, 84, 94, 104–107]. Selected individuals from published Khoe-San populations that had shown high levels of recent admixture were filtered out as described in [1]. To avoid sample-size bias in further analyses, we randomly downsized each comparative population to a maximum of 20 individuals (applying the “shuf” command in bash). The comparative data were filtered following the same quality control criteria as described above, and after merging, the final dataset was composed of 1811 samples with 305,417 overlapping autosomal SNPs. The merged dataset was phased with fastPHASE v.1.4.0 [108]. The number of haplotype clusters was set to 25 and we use 25 runs of the EM algorithm to generate the “best” haplotype guess. For the demographic model testing (see below), the current dataset was complemented with additional African aDNA samples (Additional File 2: Table S8).

The assessment of the allele frequencies for the lactase persistence mutations was performed exclusively among Hessequa descendants using PLINK on the raw data in order to control for possible errors due to flipping between forward and reverse strands while merging with other datasets.

### ADMIXTURE, local ancestry inference and admixture dating

We ran initial population structure analyses and estimated admixture fractions with ADMIXTURE [109]. The dataset was pruned using PLINK (indep-pairwise 200 25 04) before the admixture runs, resulting in 233,254 autosomal SNPs. The number of clusters, *K*, was set from 2 to 10, replicated 50 times with random seeds. The cluster-inference and visual inspection was made with Pong v.1.4.5 [110].

To identify ancestry segments and admixture time estimates, we use the MOSAIC software v. 1.3.1 [54]. We determined the ancestral state for each SNP in the dataset by identifying the allele present in the chimpanzee, gorilla and orangutan genomes. The ancestral state was only used if at least two out of the three apes carried the same allele, SNPs for which the ancestral state was unknown and if the allele is not present in humans were excluded. We also added the BbayA ancient individual [2] to this analysis since this individual does not contain East Africa/Eurasian ancestry and the quality of the data allow for diploid calling. We ran MOSAIC on a total of 254,954 autosomal SNPs and used the HapMap II recombination map. Initial inspection of MOSAIC suggested that the software is particularly sensitive to non-homogenous recipient populations, which could affect date estimate results. To minimize this effect in modeling a complex demographic scenario (5-way ancestry model), we generated an Hessequa descendants “meta-group” where only individuals that followed the average proportions ± one standard deviation for the five ancestries in the admixture analysis at *K* = 5, were kept. The filtering strategy is meant to minimize the impact of admixture in recent generations (last 2-to-3 generations), resulting, for example, from increased mobility and the imposed social restructuring during Apartheid. However, the same analysis was performed on each separate Hessequa descendant group for support information (Additional File 1: Figure S20-S29).

For each tested population group, we performed MOSAIC analyses in accordance with the number of sources informed by ADMIXTURE runs. For the Hessequa descendants, we ran MOSAIC under a 5-way admixture model without providing any parental source information. The Khoe-San populations, Khwe, Xade and !Xuun, were analyzed under a 3-way admixture model and the ǂKhomani, Nama Windhoek and Nama Richtersveld under a 4-way model. We bootstrapped individuals for the pairwise co-ancestry curves estimates 100 times to obtain the admixture timing intervals.

We confirmed the MOSAIC results for the Hessequa descendants, with population model testing in the 58 Hessequa descendants, using the qpGraph package (ADMIXTOOLS, [111]). To minimize the impact of admixture in the model, qpGraph was calculated using hunter-gatherer aDNA from southern Africa (approx. 2000 years old) [2, 6], southern Africa pastoralist (approx. 1200 years old) [6] and Pastoral Neolithic samples from Kenya and Tanzania (approx. 2500 years old) [84]. To complement the ancient samples, we also included modern-day YRI, CEU and GIH [104] to capture the five main ancestries present in the Hessequa descendant gene pool. Over 80 alternative models were tested and only the presented result could not be discarded.

### X chromosome/autosomal ancestries proportion ratio

To test whether the arrival of the East African component in southern Africa was sex-biased, we estimated the X chromosome/Autosomal ratio for admixture proportions. The tested populations were merged with a comparative dataset of 20 Yoruba (YRI), 20 Amhara, 20 Central Europeans (CEU), 20 Sri Lankan Tamil (STU) and 17 Ju|’hoan, which were used in the supervised admixture to have known ancestries. X chromosomes from both males and females were included and the data was filtered using the same criteria settings as the autosomes described above, excluding HWE filtering.

To avoid discrepancies in resolution power between the X chromosome and autosome sizes, we selected chromosomes 7, 10 and 12 since they have approximately the same genetic length as the X chromosome (180 cM). Furthermore, we cut the first 180 cM of chromosomes 1 to 6 as well. For each of the selected autosomes, we randomly down sampled SNPs to the same number present in X chromosome. The “shuf” command in bash was applied for random down-sampling. We ran supervised admixture for chromosomes 1 to 7, 10, 12 and X separately, replicated 20 times each. The cluster-inference and visual inspection was made with Pong v.1.4.5 [110], and averaged per population ancestry proportions were noted. Only Khoe-San groups that hold an average East Africa ancestry higher than 2% in the autosomes and X chromosome (Hessequa descendants, Nama from Windhoek, Khwe, ǂKhomani and Coloured population of Wellington) were selected for subsequent X chromosome/autosomal ratios. This criterion was applied to avoid spurious results (if a group has minimal ancestry fraction, then it can disappear from a specific chromosome more easily due to chance, which inflates/deflates the ratios dramatically). The X chromosome/autosomal ratios were calculated between the X chromosome and each specific (trimmed) autosome independently and averaged across estimations. The X chromosome to autosomal ratio was performed with random sampling bootstraps and average and standard deviations were calculated. To evaluate if the X chromosome/autosomal ratio of the selected Hessequa descendants were influenced by the down-sampling, the ratios were also calculated on the whole dataset and presented per sampling group (Additional File 1: Figure S31).

### Mitochondrial DNA (mtDNA)

We selected 7–12 Hessequa descendants randomly from the nine sampling sites (in accordance with sample availability), resulting in 87 individuals to sequence the complete mtDNA: Heidelberg (*N* = 10), Melkhoutfontein (*N* = 10), Railton (*N* = 8), Riversdale (*N* = 12), Rotterdam Farm (*N* = 7), Slangrivier (*N* = 8), Stormsvlei (*N* = 10), Suurbraak (*N* = 11) and Swellendam (*N* = 11). We amplified two fragments of 7.7 kb and 9.2 kb (which cover the full mitochondrial genome), following the protocol proposed by [112]. The two fragments were pooled equimolarly and each sample was uniquely barcoded. The samples were sent to the SciLifeLab in Uppsala and sequenced on the PacBioRSII. The two sequenced fragments were merged into one full-length mtDNA sequence for each individual. The full mtDNA sequence was aligned to the Revised Cambridge Reference Sequence (rCRS) to create consensus BAM files. The samples were converted to FASTA files using SAMTOOLS version 1.9 [113]. Average sequencing coverage was determined for all samples (using samtools depth). Mitochondrial haplogroups were assigned using HaploGrep2 [114]. Ancestries of mtDNA haplogroups (Additional File 2: Table S7) were assigned according to population groups in which these haplogroups were highly prevalent in previously published studies [57–59, 65, 115].

### SNAPPY analysis for determination of the Y haplogroup

For each self-identified male in the Hessequa descendant groups (*N* = 58), Y chromosome-specific markers were extracted, and 2538 SNPs were retained. The number of males (*N* = 58) were not the same individuals that were selected after recent admixture removal (also *N* = 58) nor were they the same individuals carrying the L0d mtDNA haplogroup (also *N* = 58)—the re-occurrence of the number 58 for these individual groupings was a chance event. Y chromosomal markers were used to assign Y haplogroups using SNAPPY [116]. Haplogroup assignment was calculated for all males in the dataset, resulting in haplogroup assignments where the accuracy score was above 75% for all assignments. Ancestries of Y chromosome haplogroups (Additional File 2: Table S7) were assigned according to population groups in which these haplogroups were highly prevalent in previously published studies [57, 64, 65, 117–119].

## Supplementary information

**Additional file 1: **PDF file with supplementary figures: **Figure S1.** Distribution of Cape Khoekhoe groups along the Cape west, south and southeast coasts. **Figure S2.** ADMIXTURE clustering analysis. The analyses are based on 1811 samples with 233,254 overlapping autosomal SNPs. **Figure S3.** ADMIXTURE clustering analysis zoom-in for Hessequa-descendants and Coloured groups across K2-10. **Figure S4.** ADMIXTURE cross-validation error. **Figure S5.** Demographic model testing for the Hessequa-descendants using qpGraph. **Figure S 6.** 1–F_ST_ estimates between local ancestral groups of the Hessequa-descendants and each donor panel. **Figure S7–S12.** 1–F_ST_ estimates between local ancestral groups of the each Khoe-San comparative group and their donor panels. **Figure S13.** Inferred pairwise coancestry curves in the Hessequa-descendants under a 5-way admixture model without pre-defining reference panel. **Figure S14.** Pairwise coancestry curves in the Hessequa-descendants under a 3-way admixture model using a reference panel. **Figure S15–S20.** Inferred pairwise coancestry curves for each comparative group under their best admixture model. **Figure S21–S29.** 1–F_ST_ estimates between local ancestral groups of each Hessequa-descendants sampling site and each donor panel. **Figure S30.** Supervised ADMIXTURE clustering analysis for the X-chromosome at K = 5. **Figure S31.** X-chromosome to autosomal ratio for each Hessequa-descendants sampling site.

**Additional file 2: **Excel file with supplementary tables: **Table S1.** Internal classification of Southern African Khoisan linguistic groups. **Table S2.** East African ADMIXTURE proportions in relation to East Africa and autochthonous San ancestries (i.e. excluding recent admixture from West Africans and Eurasians). **Table S3.** Allele frequencies of LP associated variants present in the H3Africa SNP array. **Table S4.** Relevant admixture time estimates based on pairwise coancestry curves. **Table S5.** X-chromosome to autosomal ratio based on the average ancestry proportion. **Table S6.** Summary results for the mitochondrial haplogroup assignment for the 87 sub-sampled Hessequa-descendants. **Table S7.** Summary results for the Ychr haplogroup assignment for the 58 male Hessequa-descendants. **Table S8.** List of populations included in the dataset.

## Data Availability

All data generated or analyzed during this study are included in this published article, its supplementary information files and publicly available repositories. The generated genotype data is available for academic research use through the European Genome-Phenome Archive [120] with accession number EGAD00010002113 (Genome-wide data, 162 individuals) and EGAD00001007676 (mtDNA sequences, 87 individuals).
