# Supplementary material for: Male-biased migration from East Africa introduced pastoralism into southern Africa
Source: BMC Biol. 2021 Dec 7;19:259. doi: 10.1186/s12915-021-01193-z (PMC8650298; doi:10.1186/s12915-021-01193-z)
Supplement: Supplementary file 1 — Additional file 1: PDF file with supplementary figures: Figure S1. Distribution of Cape Khoekhoe groups along the Cape west, south and southeast coasts. Figure S2. ADMIXTURE clustering analysis. The analyses are based on 1811 samples with 233,254 overlapping autosomal SNPs. Figure S3. ADMIXTURE clustering analysis zoom-in for Hessequa-descendants and Coloured groups across K2-10. Figure S4. ADMIXTURE cross-validation error. Figure S5. Demographic model testing for the Hessequa-descendants using qpGraph. Figure S 6. 1–FST estimates between local ancestral groups of the Hessequa-descendants and each donor panel. Figure S7–S12. 1–FST estimates between local ancestral groups of the each Khoe-San comparative group and their donor panels. Figure S13. Inferred pairwise coancestry curves in the Hessequa-descendants under a 5-way admixture model without pre-defining reference panel. Figure S14. Pairwise coancestry curves in the Hessequa-descendants under a 3-way admixture model using a reference panel. Figure S15–S20. Inferred pairwise coancestry curves for each comparative group under their best admixture model. Figure S21–S29. 1–FST estimates between local ancestral groups of each Hessequa-descendants sampling site and each donor panel. Figure S30. Supervised ADMIXTURE clustering analysis for the X-chromosome at K = 5. Figure S31. X-chromosome to autosomal ratio for each Hessequa-descendants sampling site. [file 12915_2021_1193_MOESM1_ESM.pdf]

Supplemental information

Male-biased migration from East Africa introduced pastoralism into southern Africa

Mário Vicente[1,2,3], Imke Lankheet[1], Thembi Russell[4], Nina Hollfelder[1], Vinet Coetzee[5], Himla Soodyall[6,7], Michael De Jongh[8] and Carina M. Schlebusch[1,9,10*]

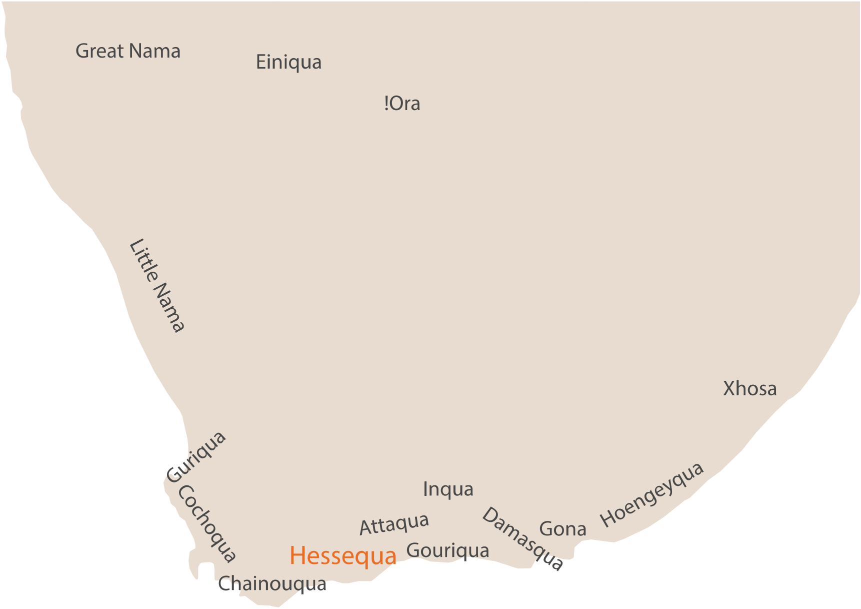

Figure S 1 – Distribution of Cape Khoekhoe groups along the Cape west, south and southeast coasts. Einiqua and !Ora (Korana) are other Khoekhoe languages, spoken inland along river valleys e.g. the Gariep river. Xhosa is a Bantu-language. (Adapted from Gilmore and Mbenga (2007) and De Jongh 2016).

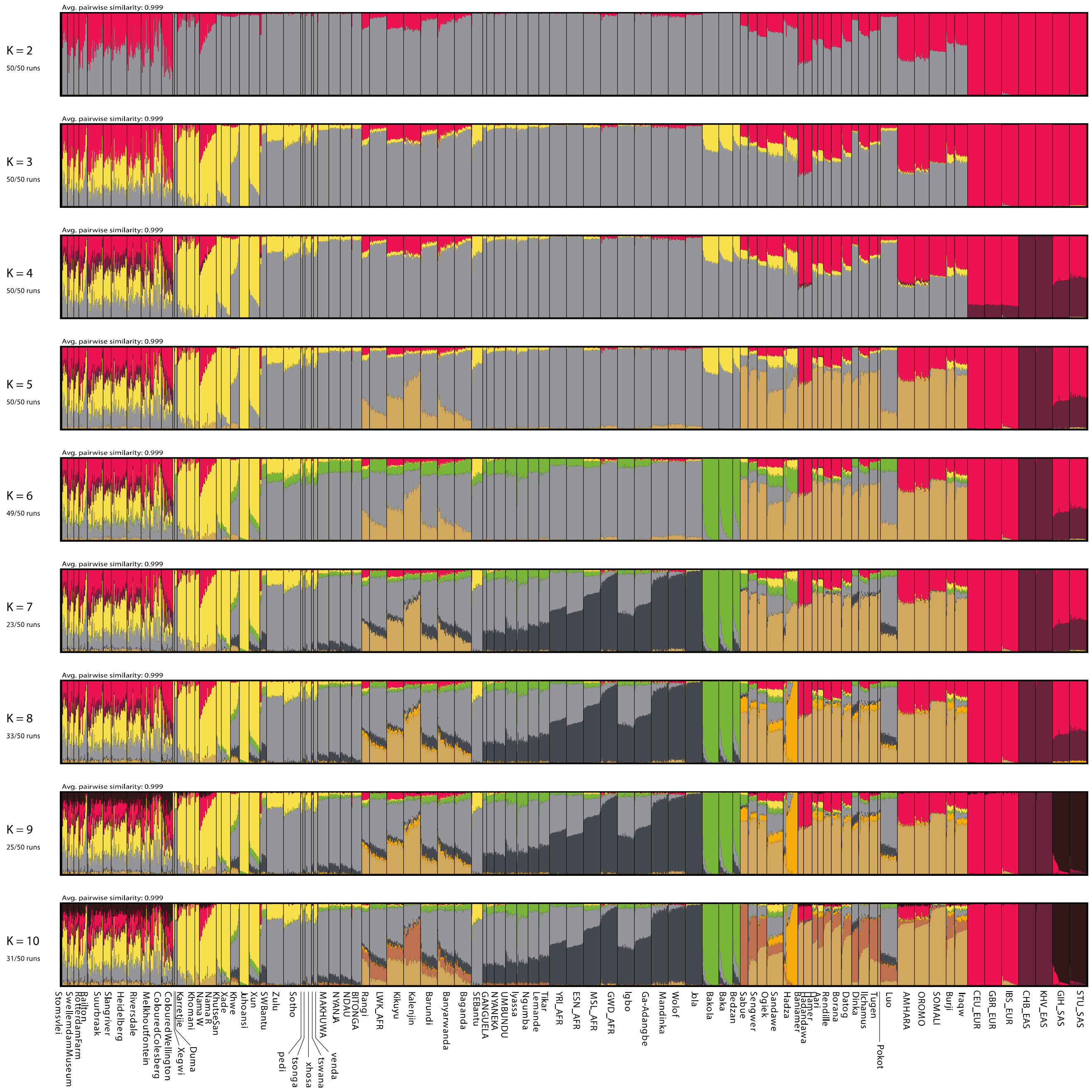

Figure S 2 - ADMIXTURE clustering analysis. The analyses are based on 1811 samples with 233,254 overlapping autosomal SNPs. Fifty independent runs were performed with different random seeds, for each K. The number of iterative runs that support each specific K cluster assignment are given on the left of the figure.

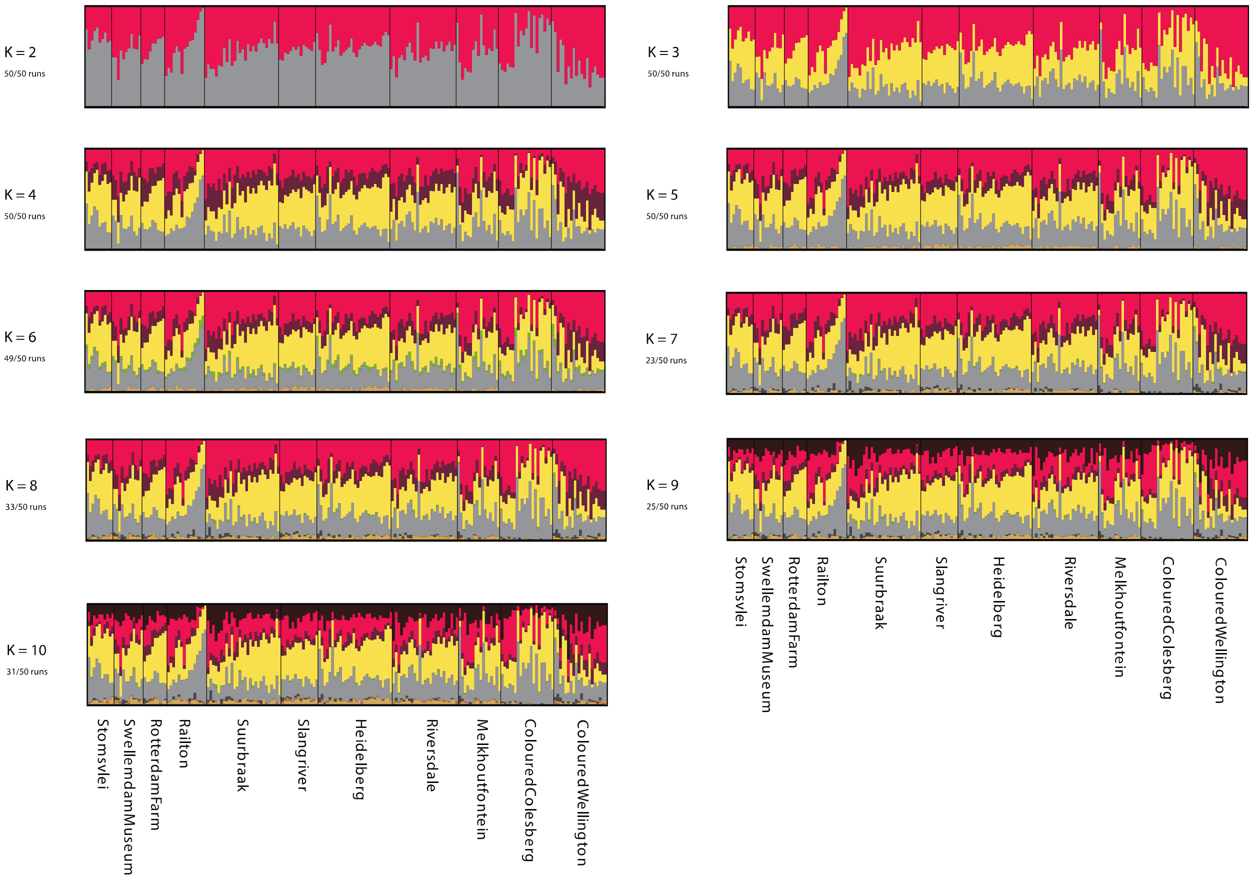

Figure S 3 –ADMIXTURE clustering analysis zoom-in for Hessequa-descendants and Coloured groups across K2-10. Fifty independent runs were performed with different random seeds, for each K. The number of iterative runs that support each specific K cluster assignment are given on the left of the figure.

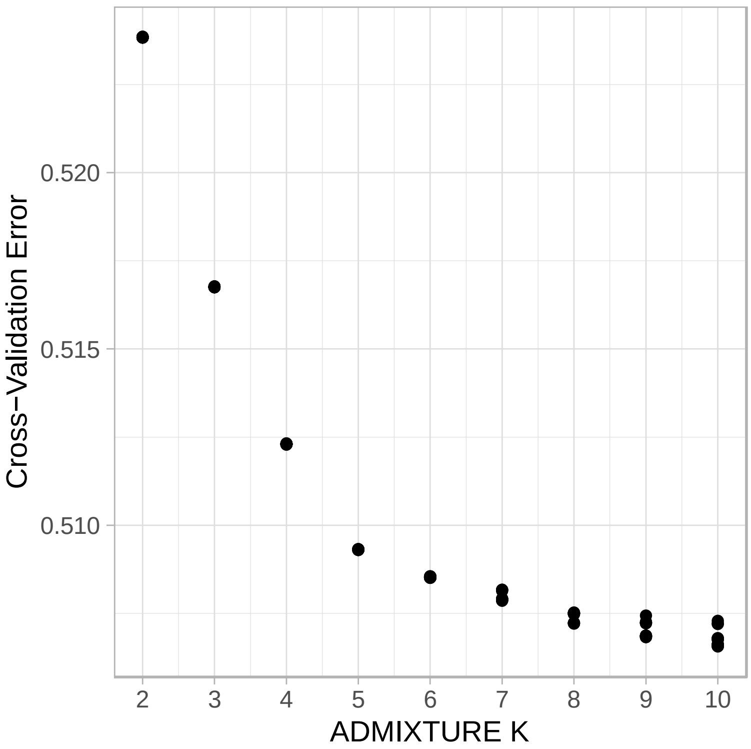

Figure S 4 - ADMIXTURE cross-validation error. Fifty independent runs were performed with different random seeds, for each K.

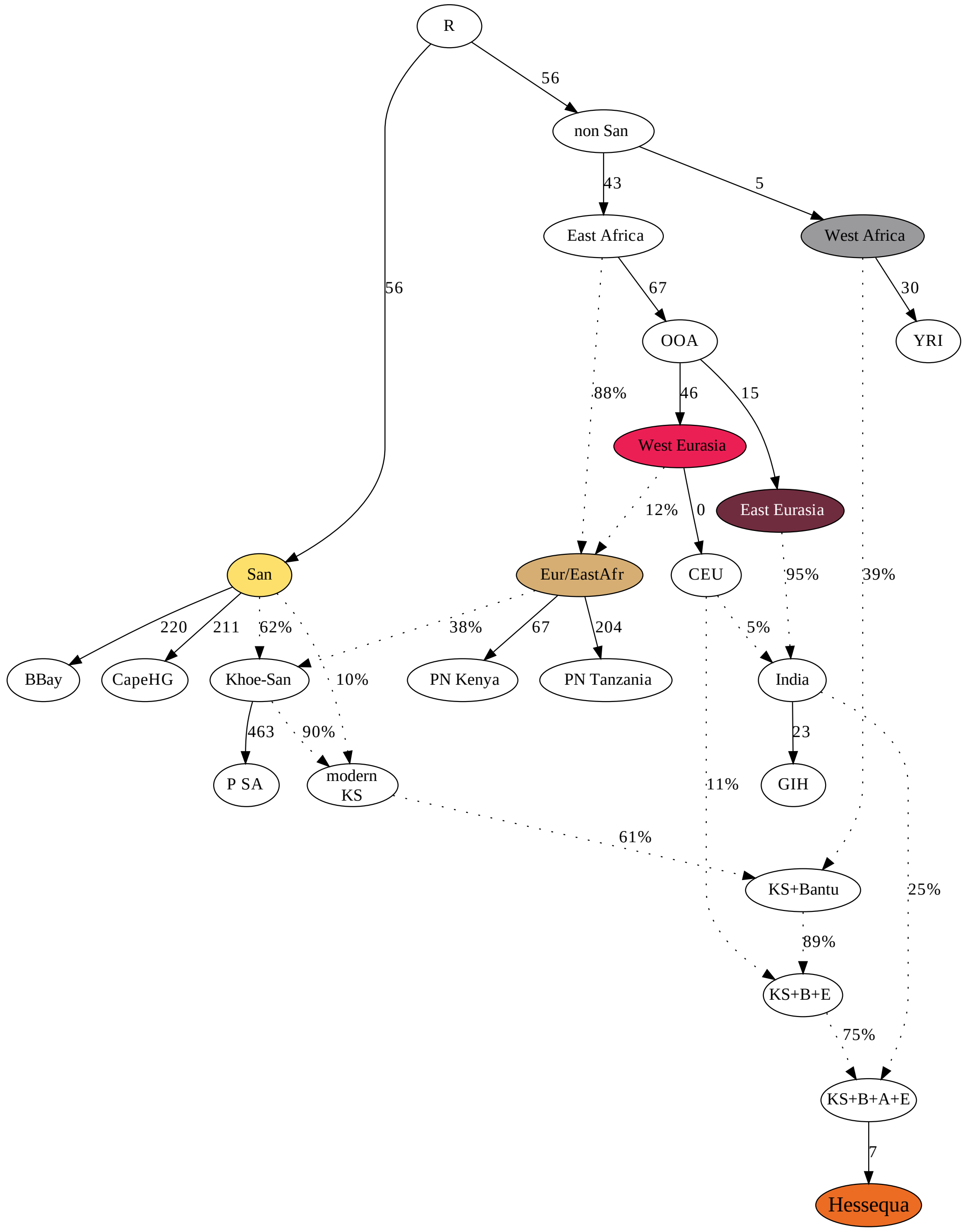

Figure S 5 – Demographic model testing for the Hessequa-descendants using qpGraph. Worst Z-score: Pastoral Neolithic Kenya (PNK), Southern Africa Pastoral Stone Age (PSA), Pastoral Neolithic Kenya (PNK), Hessequa-descendants (Hes), Z-score: 2.424 Solid arrows represent direct branching with the direction stated by the arrow. Values adjacent indicates branch length in drift units. Dotted-lines represent admixture between two populations with the proportion of the contribution. KS: Khoe-San ancestral population; B: Bantu-speaking ancestral population; A: Asian ancestral population; E: European ancestral population.

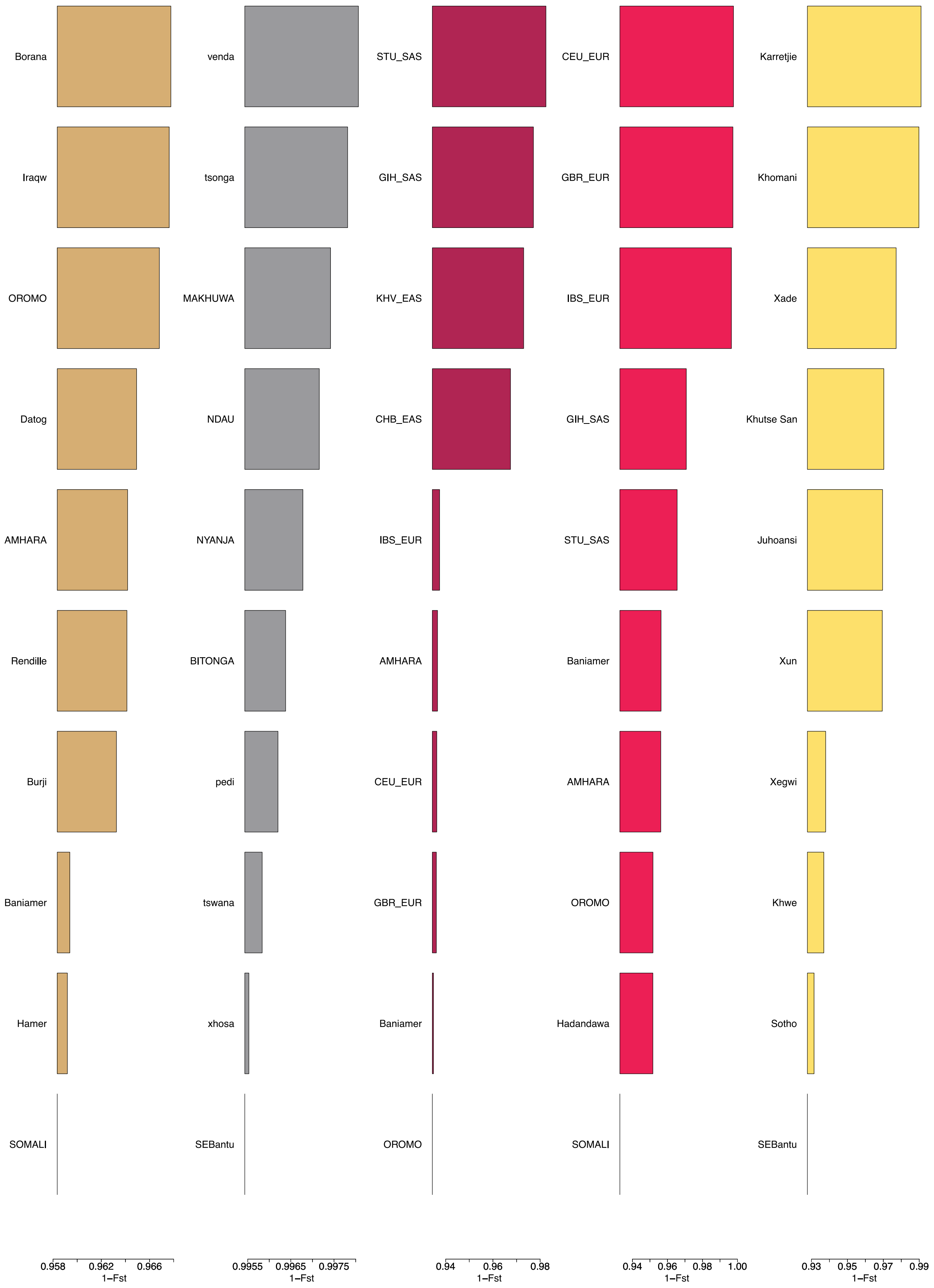

Figure S 6 – 1–F_{ST} estimates between local ancestral groups of the Hessequa-descendants and each donor panel. The values were obtained conditioned on a 5-way admixture model using MOSAIC (Salter-Townshend and Meyers 2019). To each ancestral group is reported the top 10 “1–F_{ST}” values across the populations present in the dataset (Table S8).

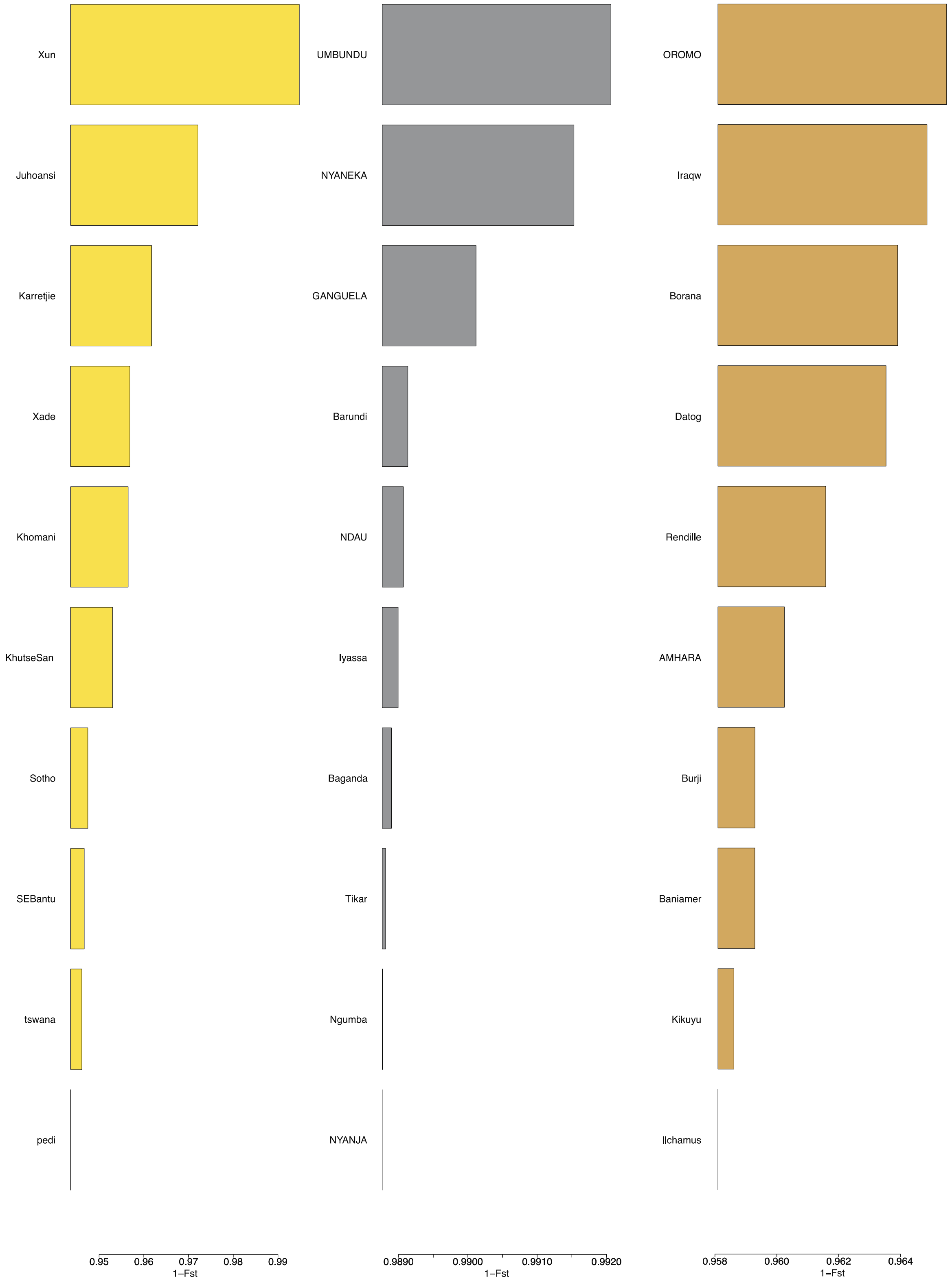

Figure S 7 – 1–F_{ST} estimates between local ancestral groups of the Khwe and each donor panel. The values were obtained conditioned on a 3-way admixture model using MOSAIC. To each ancestral group is reported the top 10 "1–F_{ST}" values across the populations present in the dataset (Table S8).

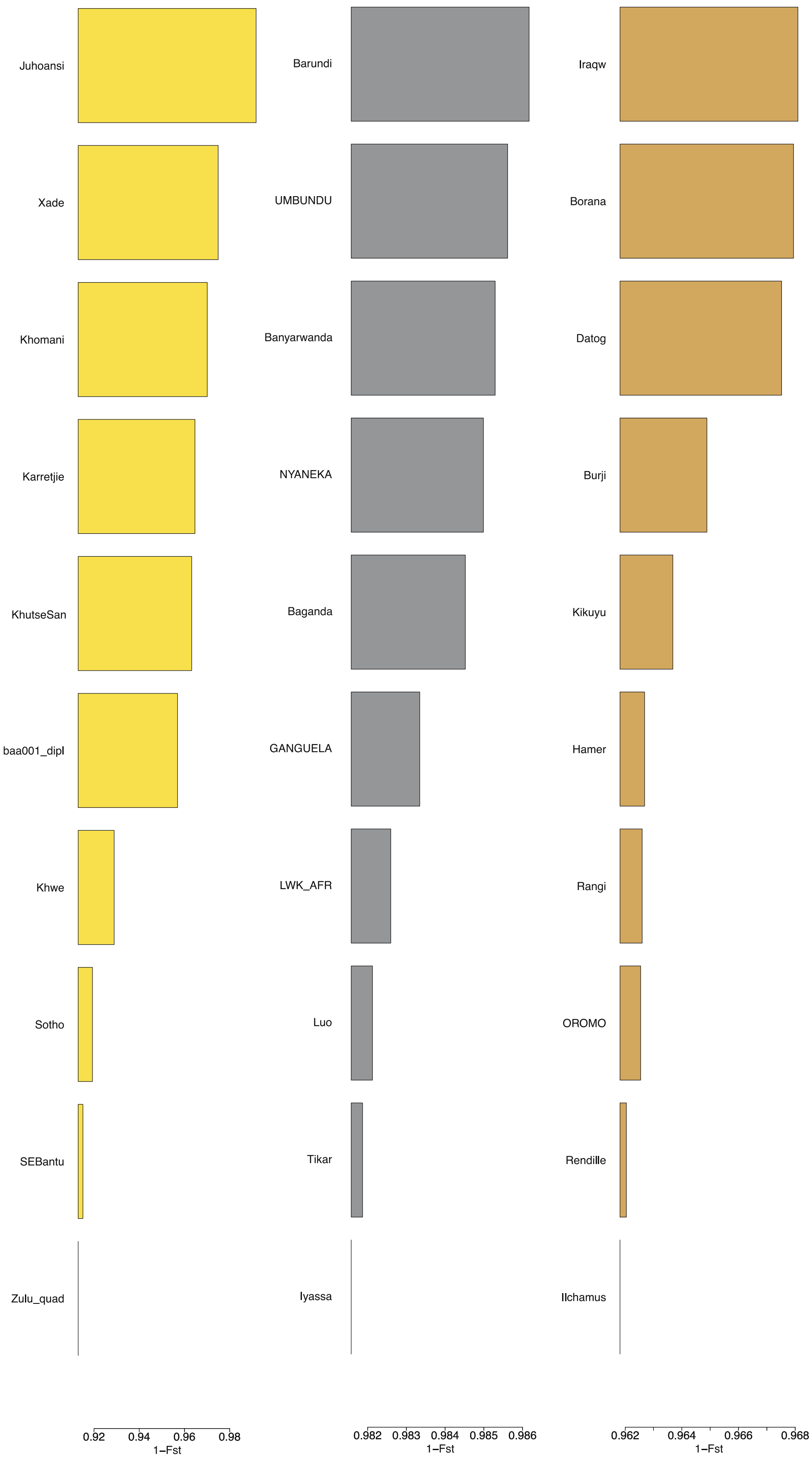

Figure S 8 – 1–F_{ST} estimates between local ancestral groups of !Xuun of Angola and each donor panel. The values were obtained conditioned on a 3-way admixture model using MOSAIC. To each ancestral group is reported the top 10 "1–F_{ST}" values across the populations present in the dataset (Table S8).

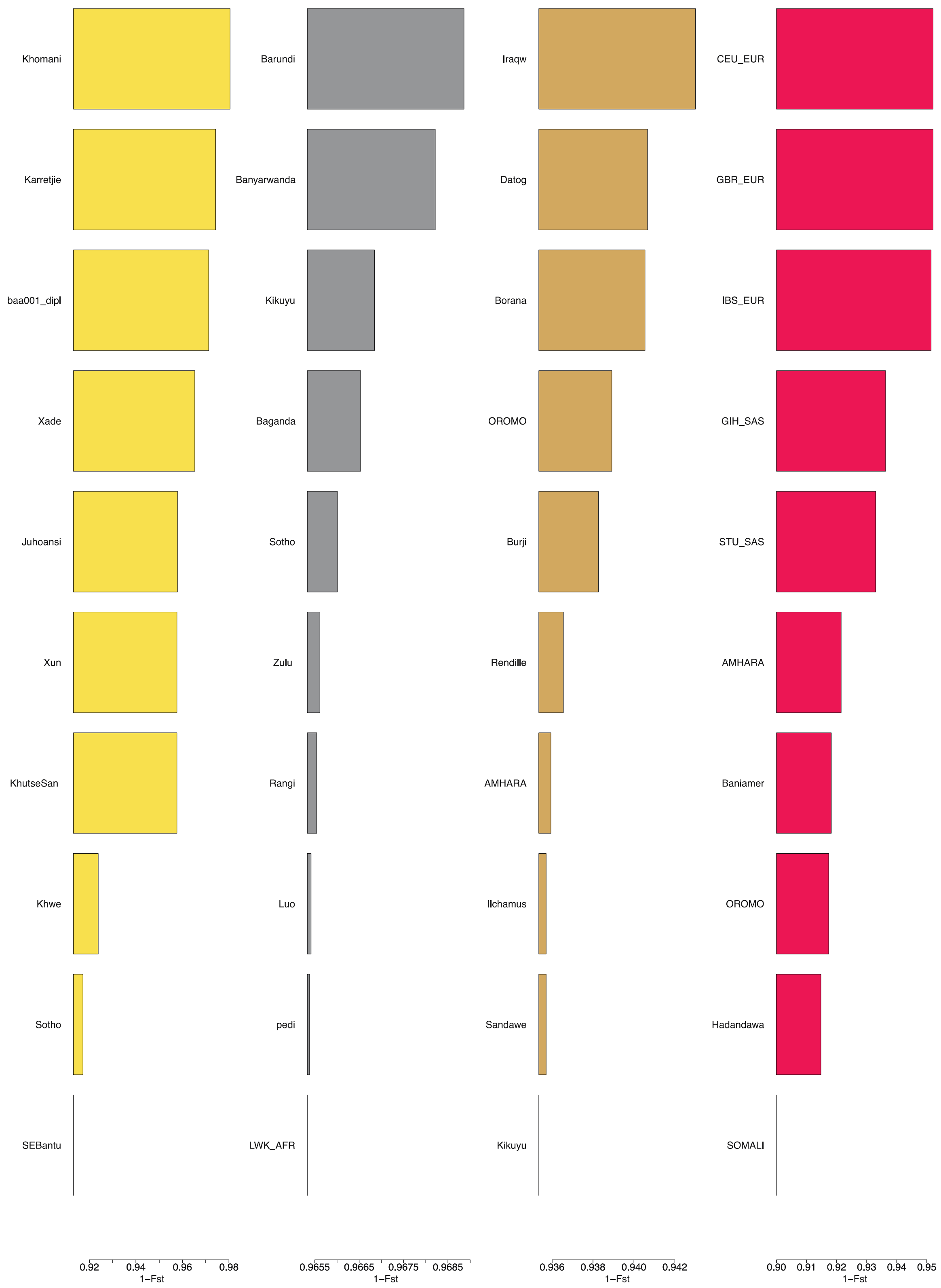

Figure S 9 – 1–F_{ST} estimates between local ancestral groups of the Nama from Richtersveld and each donor panel. The values were obtained conditioned on a 4-way admixture model using MOSAIC. To each ancestral group is reported the top 10 "1–F_{ST}" values across the populations present in the dataset (Table S8).

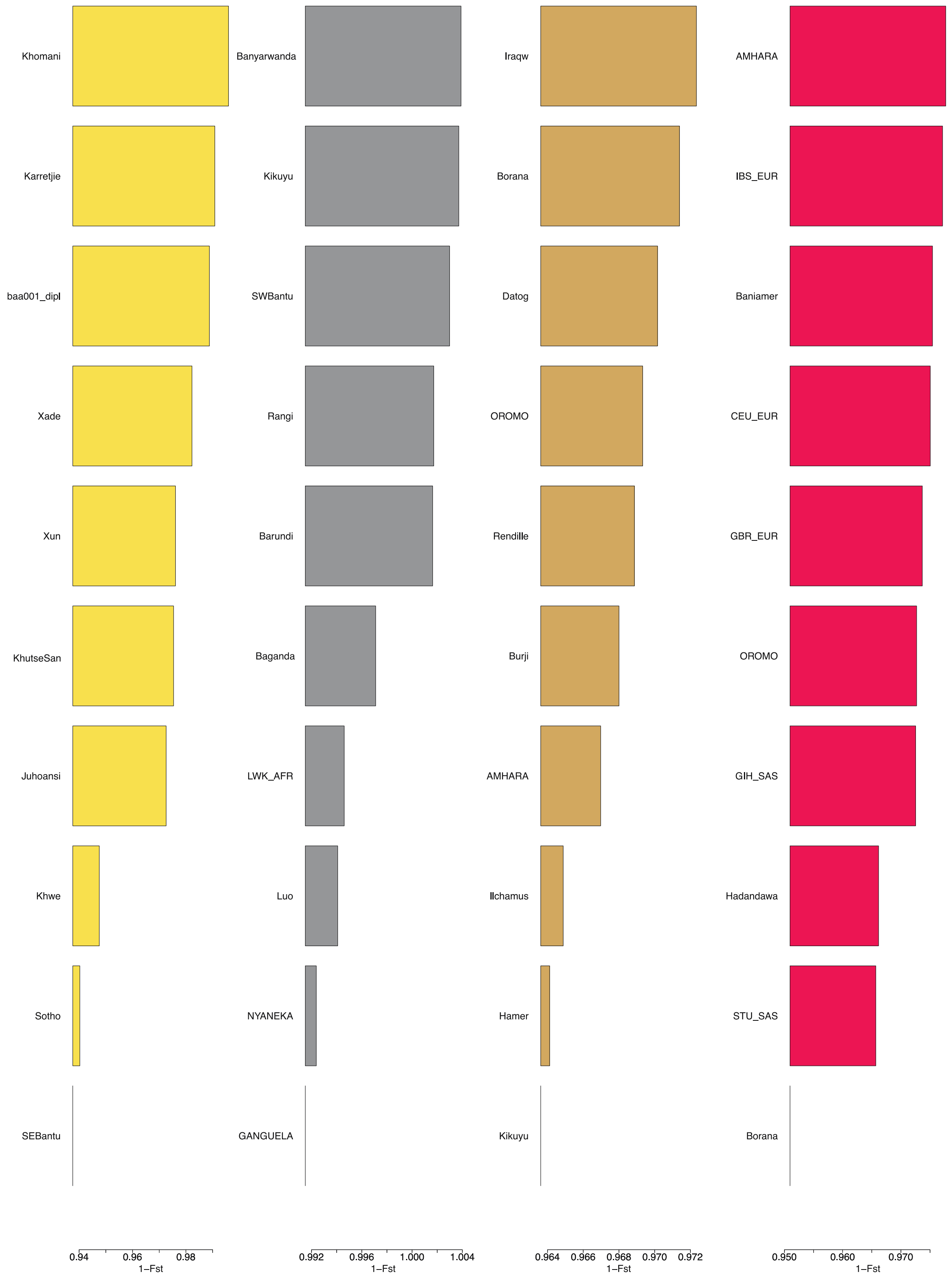

Figure S 10 – 1–F_{ST} estimates between local ancestral groups of the Nama from Windhoek and each donor panel. The values were obtained conditioned on a 4-way admixture model using MOSAIC. To each ancestral group is reported the top 10 "1–F_{ST}" values across the populations present in the dataset (Table S8).

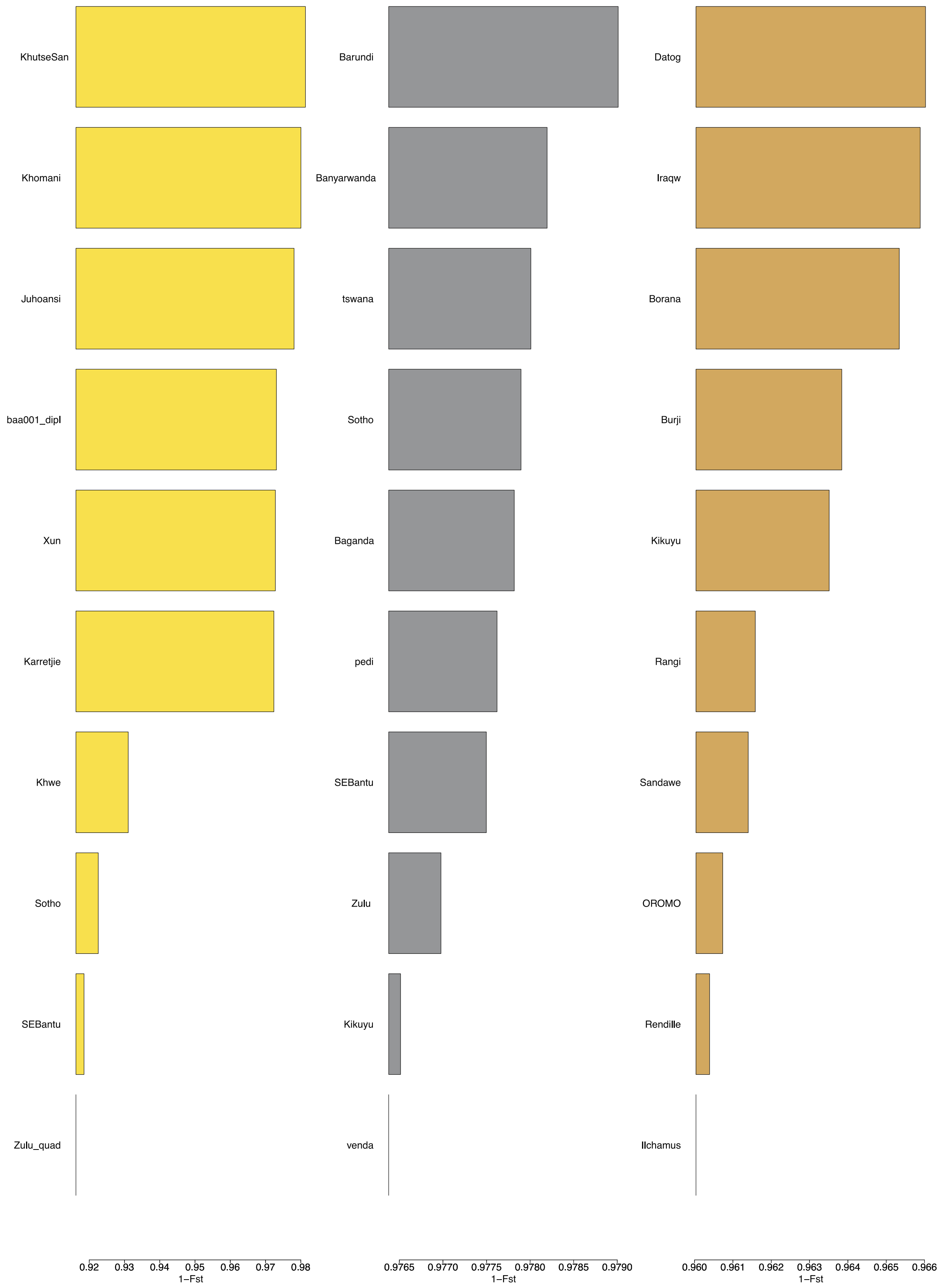

Figure S 11 – 1–F_{ST} estimates between local ancestral groups of the KhoeSan from Xade and each donor panel. The values were obtained conditioned on a 3-way admixture model using MOSAIC. To each ancestral group is reported the top 10 "1–F_{ST}" values across the populations present in the dataset (Table S8).

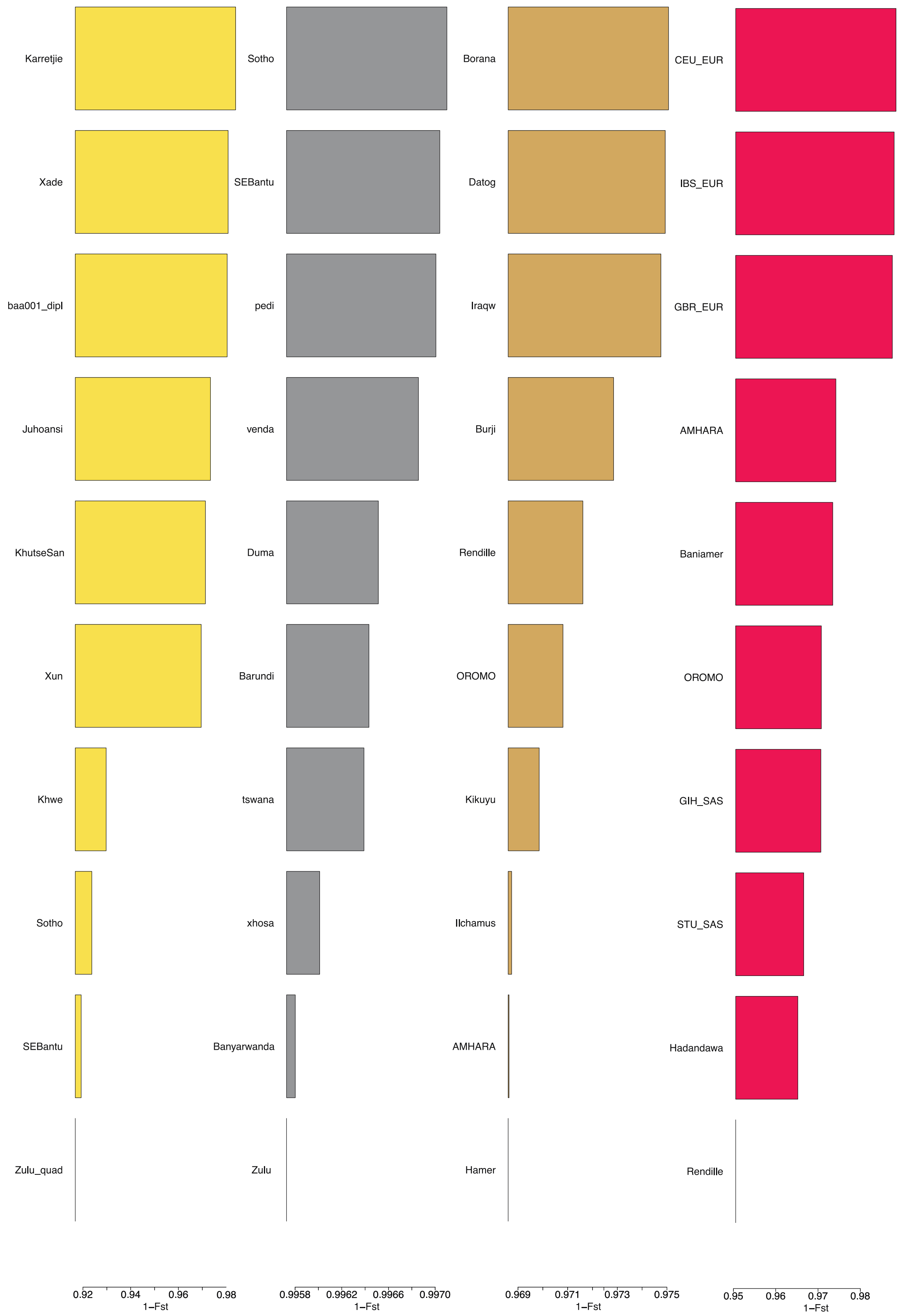

Figure S 12 – 1–F_{ST} estimates between local ancestral groups of the ǂKhomani and each donor panel. The values were obtained conditioned on a 4-way admixture model using MOSAIC. To each ancestral group is reported the top 10 "1–F_{ST}" values across the populations present in the dataset (Table S8).

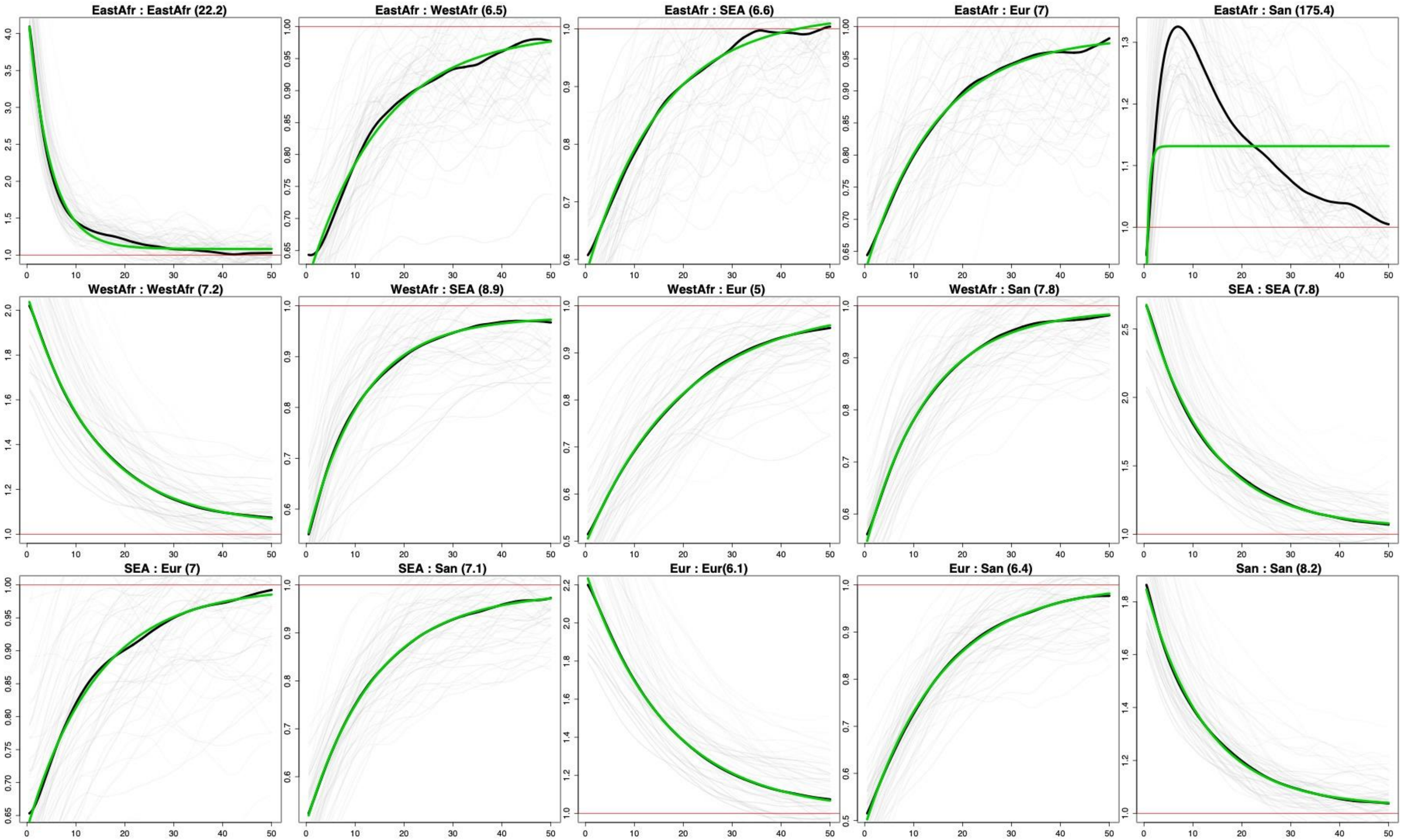

Figure S 13 – Inferred pairwise coancestry curves in the Hessequa-descendants under a 5-way admixture model without pre-defining reference panel. The date estimations use exponential decay of the ratio of probabilities for pairwise local ancestries (y-axis) as a function of genetic distance (in centi-Morgans, x-axis). The green line depicts the fitted curve, the black line the overall targets observed ratios, and the grey lines the per target ratio. The top of each panel of the pair of ancestries being examined, here stating major associated ancestry for simplicity for the reader. Within brackets is the corresponding number of generations since admixture. EastAfr: East African-related ancestry; WestAfr: West African-related ancestry; SEA: Southeast Asian-related ancestry; Eur: European-related ancestry; San: Southern Africa hunter-gatherer (San)-related ancestry.

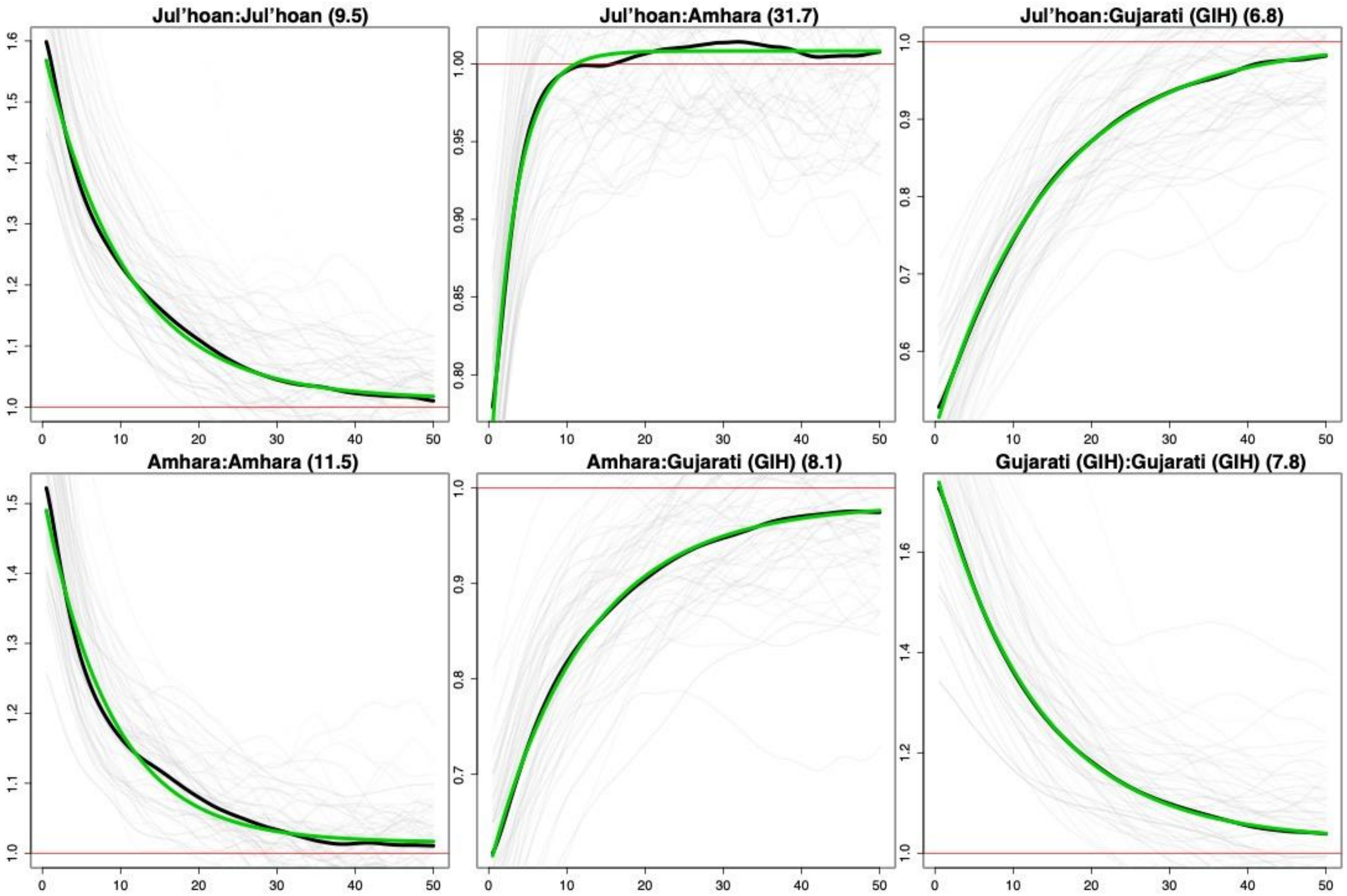

Figure S 14 – Pairwise coancestry curves in the Hessequa-descendants under a 3-way admixture model using a reference panel. The date estimations use exponential decay of the ratio of probabilities for pairwise local ancestries (y-axis) as a function of genetic distance (in centi-Morgans, x-axis). The green line depicts the fitted curve, the black line the overall targets observed ratios, and the grey lines the per target ratio. The top of each panel of the pair of ancestries being examined, here stating major associated ancestry for simplicity for the reader. Within brackets is the corresponding number of generations since admixture. San: Southern Africa hunter-gatherer (San)-related ancestry.San: Ju|'hoan, EastAfr: Amhara, Eurasian: Gujarati (GIH)

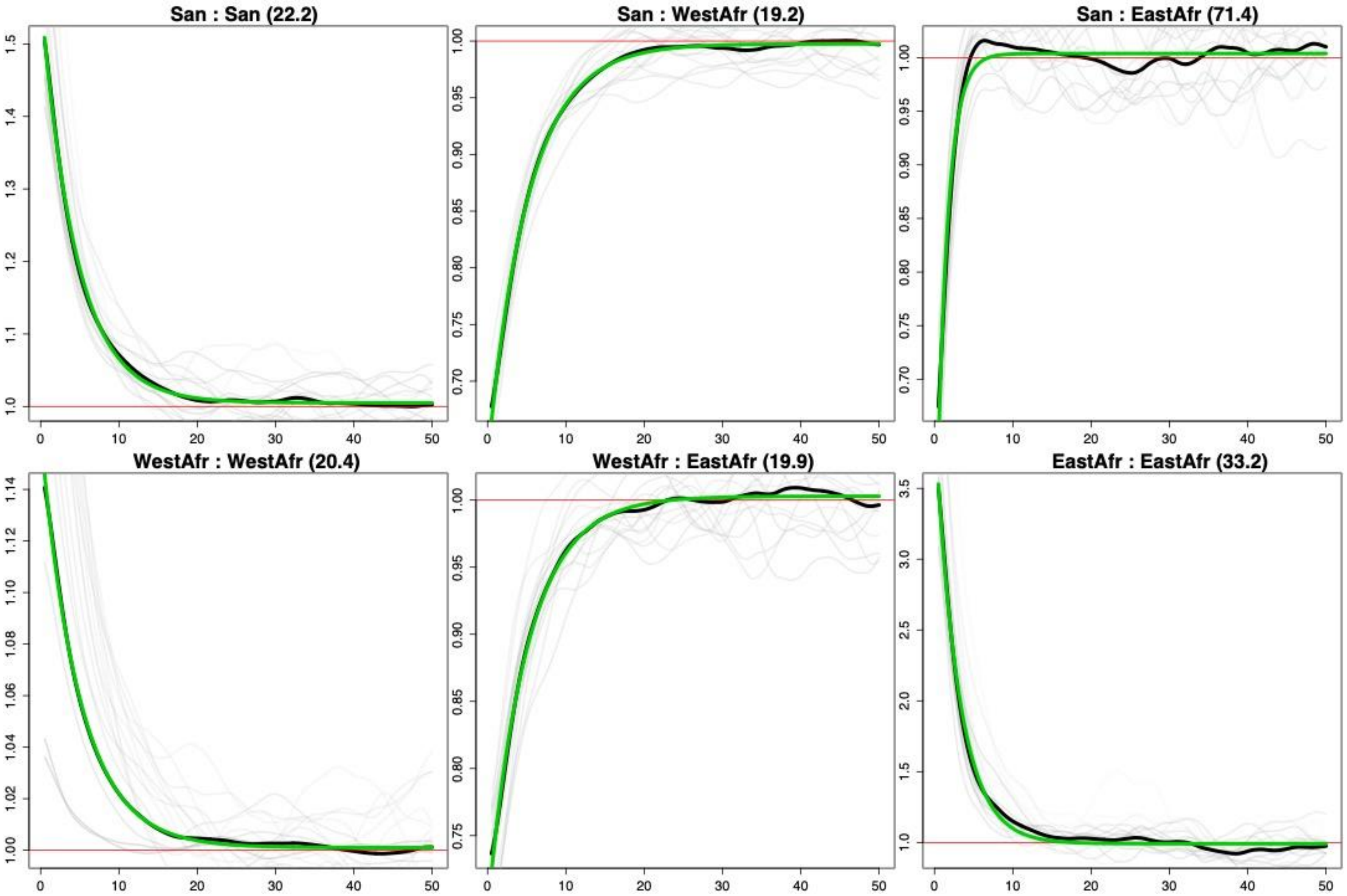

Figure S 15 – Inferred pairwise coancestry curves in the Khwe under a 3-way admixture model. The date estimations use exponential decay of the ratio of probabilities for pairwise local ancestries (y-axis) as a function of genetic distance (in centi-Morgans, x-axis). The green line depicts the fitted curve, the black line the overall targets observed ratios, and the grey lines the per target ratio. The top of each panel of the pair of ancestries being examined, here stating major associated ancestry for simplicity for the reader. Within brackets is the corresponding number of generations since admixture. EastAfr: East African-related ancestry; WestAfr: West African-related ancestry; San: Southern Africa hunter-gatherer (San)-related ancestry.

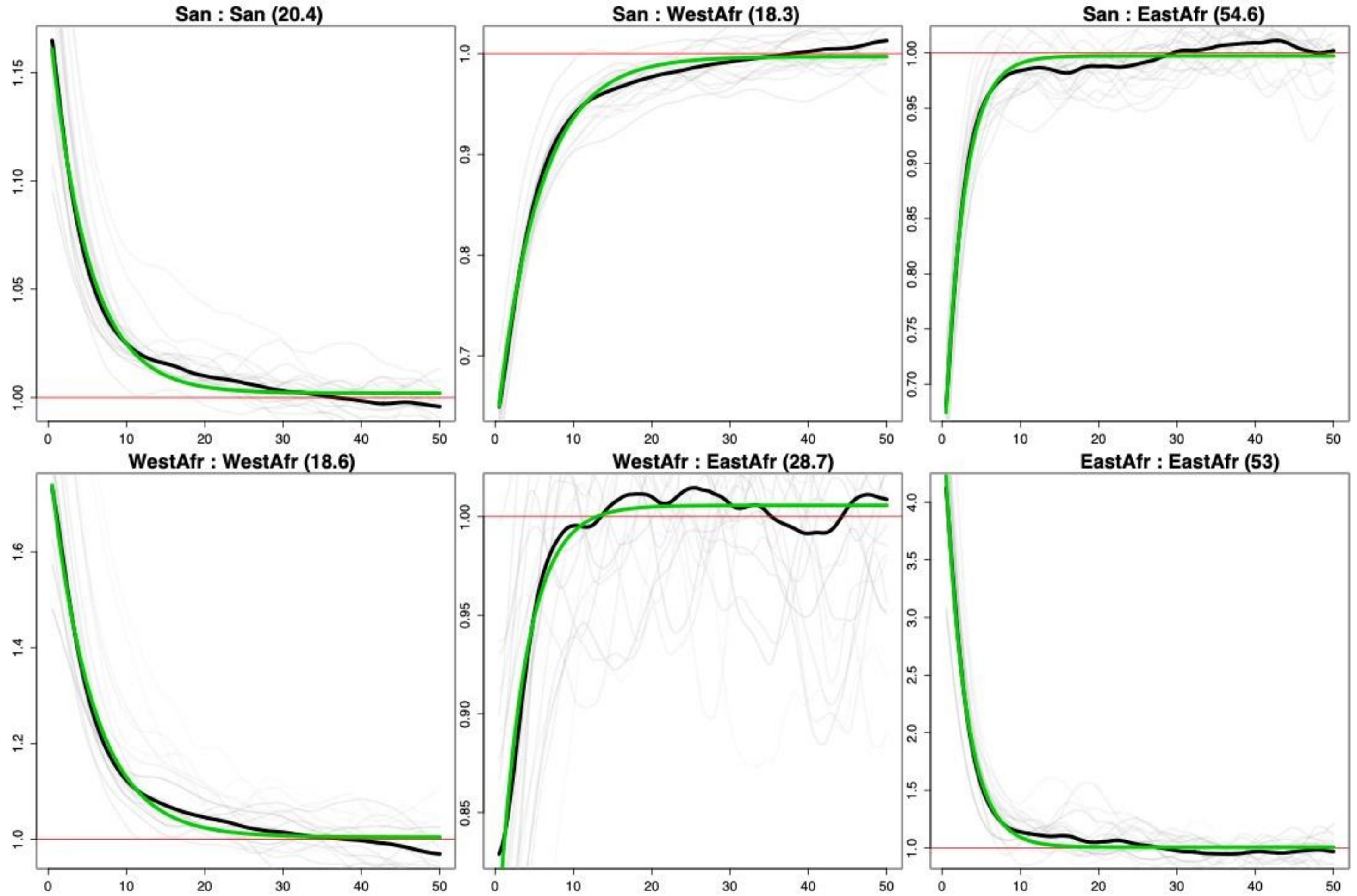

Figure S 16 – Inferred pairwise coancestry curves in the !Xuun under a 3-way admixture model. The date estimations use exponential decay of the ratio of probabilities for pairwise local ancestries (y-axis) as a function of genetic distance (in centi-Morgans, x-axis). The green line depicts the fitted curve, the black line the overall targets observed ratios, and the grey lines the per target ratio. The top of each panel of the pair of ancestries being examined, here stating major associated ancestry for simplicity for the reader. Within brackets is the corresponding number of generations since admixture. EastAfr: East African-related ancestry; WestAfr: West African-related ancestry; San: Southern Africa hunter-gatherer (San)-related ancestry.

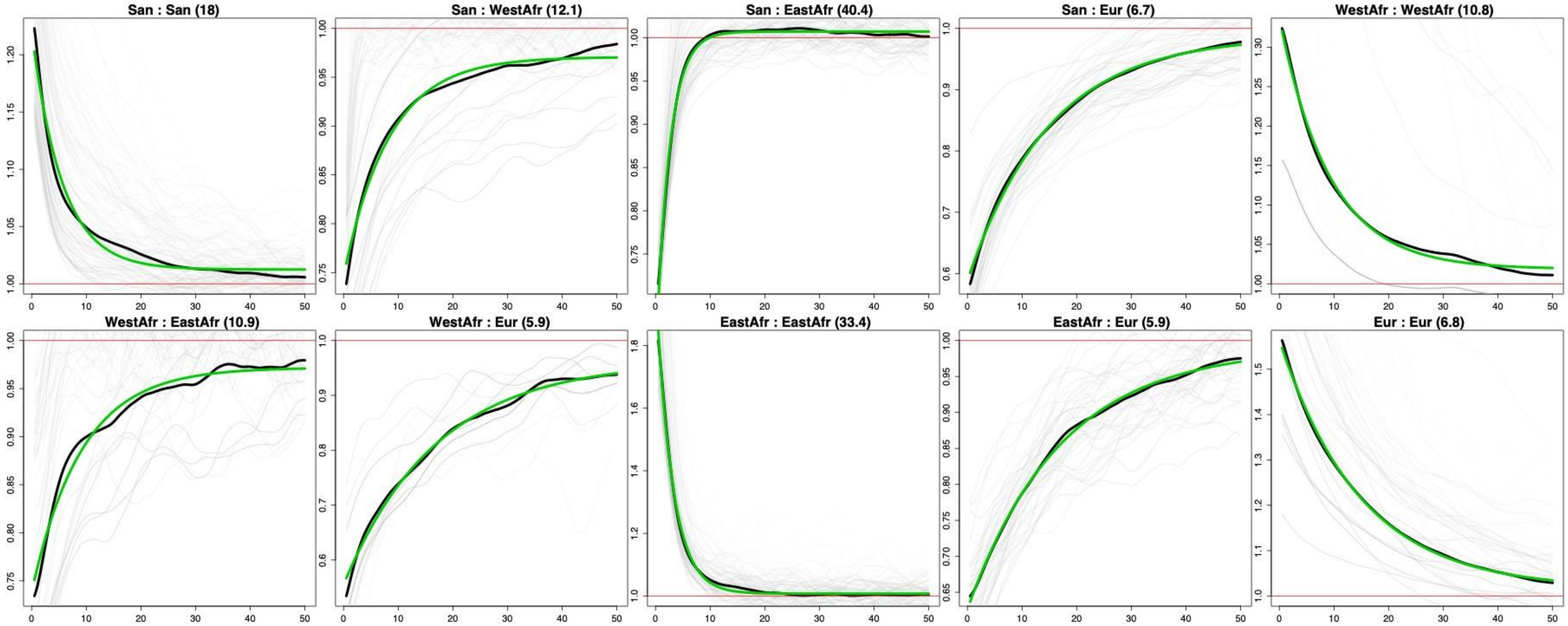

Figure S 17 – Inferred pairwise coancestry curves in the Nama from Richtersveld under a 4-way admixture. The date estimations use exponential decay of the ratio of probabilities for pairwise local ancestries (y-axis) as a function of genetic distance (in centi-Morgans, x-axis). The green line depicts the fitted curve, the black line the overall targets observed ratios, and the grey lines the per target ratio. The top of each panel of the pair of ancestries being examined, here stating major associated ancestry for simplicity for the reader. Within brackets is the corresponding number of generations since admixture. EastAfr: East African-related ancestry; WestAfr: West African-related ancestry; Eur: European-related ancestry; San: Southern Africa hunter-gatherer (San)-related ancestry.

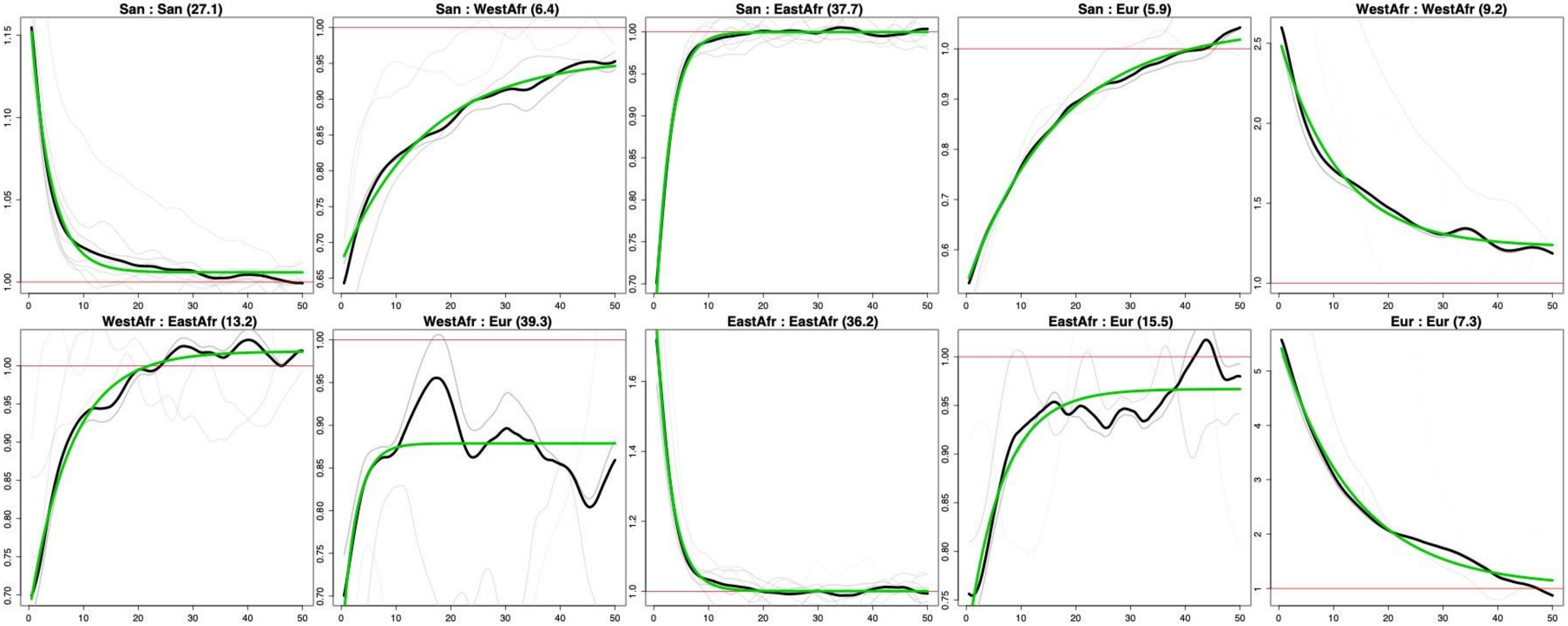

Figure S 18 – Inferred pairwise coancestry curves in the Nama from Windhoek under a 4-way admixture model. The date estimations uses exponential decay of the ratio of probabilities for pairwise local ancestries (y-axis) as a function of genetic distance (in centi-Morgans, x-axis). The green line depicts the fitted curve, the black line the overall targets observed ratios, and the grey lines the per target ratio. The top of each panel of the pair of ancestries being examined, here stating major associated ancestry for simplicity for the reader. Within brackets is the corresponding number of generations since admixture. EastAfr: East African-related ancestry; WestAfr: West African-related ancestry; Eur: European-related ancestry; San: Southern Africa hunter-gatherer (San)-related ancestry.

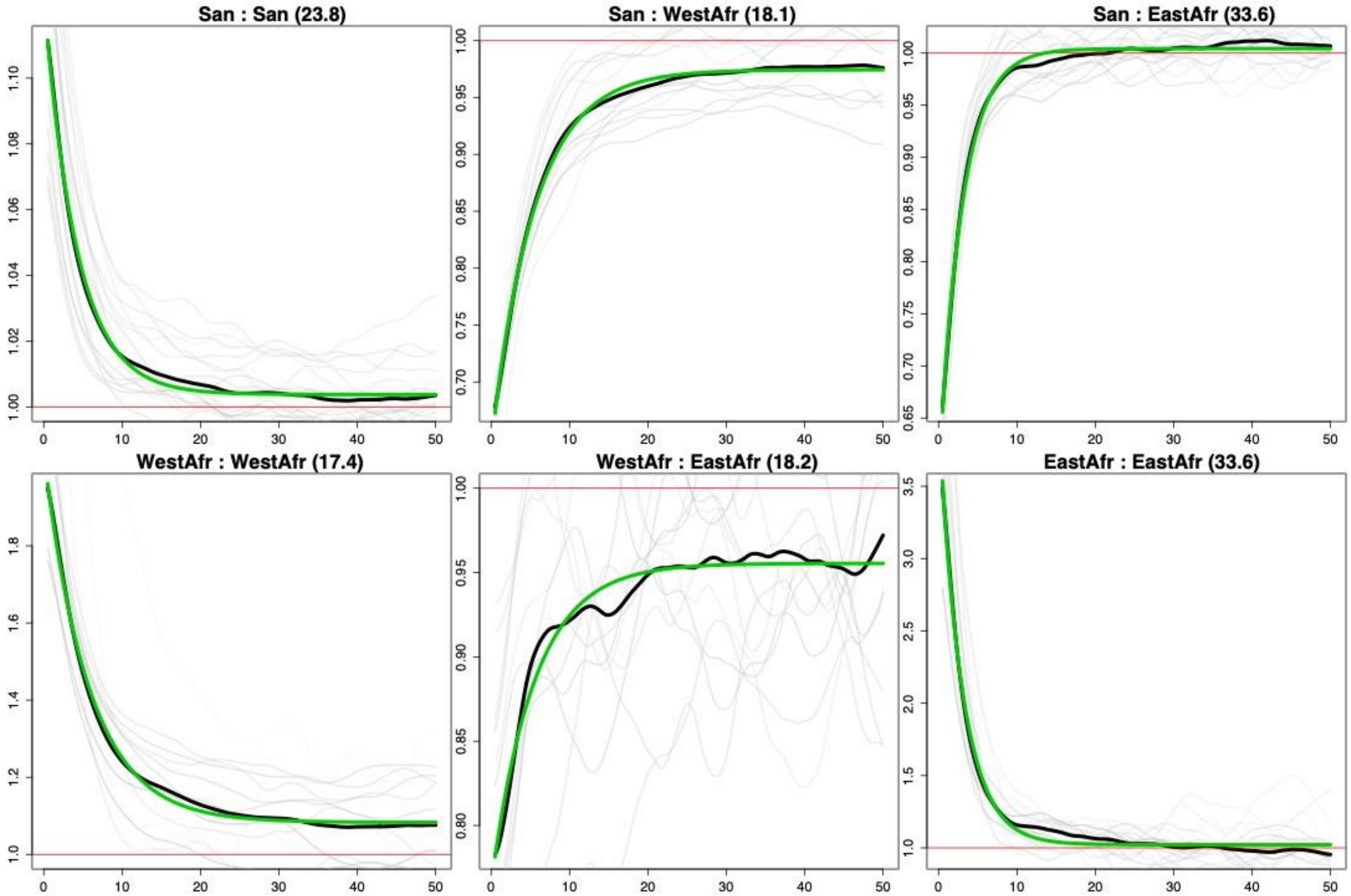

Figure S 19 – Inferred pairwise coancestry curves in the San from Xade under a 3-way admixture model. The date estimations uses exponential decay of the ratio of probabilities for pairwise local ancestries (y-axis) as a function of genetic distance (in centi-Morgans, x-axis). The green line depicts the fitted curve, the black line the overall targets observed ratios, and the grey lines the per target ratio. The top of each panel of the pair of ancestries being examined, here stating major associated ancestry for simplicity for the reader. Within brackets is the corresponding number of generations since admixture. EastAfr: East African-related ancestry; WestAfr: West African-related ancestry; San: Southern Africa hunter-gatherer (San)-related ancestry.

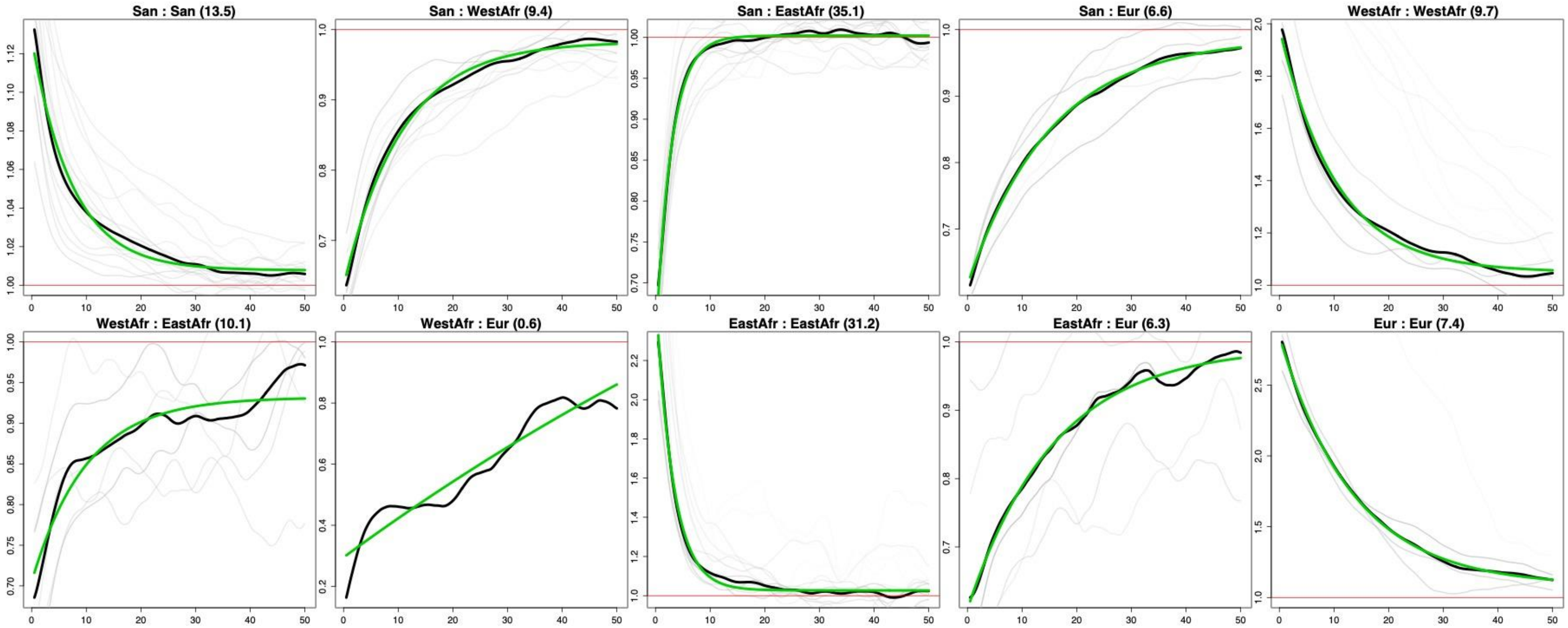

Figure S 20 – Inferred pairwise coancestry curves in the ǂKhomani under a 4-way admixture model. The date estimations use exponential decay of the ratio of probabilities for pairwise local ancestries (y-axis) as a function of genetic distance (in centi-Morgans, x-axis). The green line depicts the fitted curve, the black line the overall targets observed ratios, and the grey lines the per target ratio. The top of each panel of the pair of ancestries being examined, here stating major associated ancestry for simplicity for the reader. Within brackets is the corresponding number of generations since admixture. EastAfr: East African-related ancestry; WestAfr: West African-related ancestry; Eur: European-related ancestry; San: Southern Africa hunter-gatherer (San)-related ancestry.

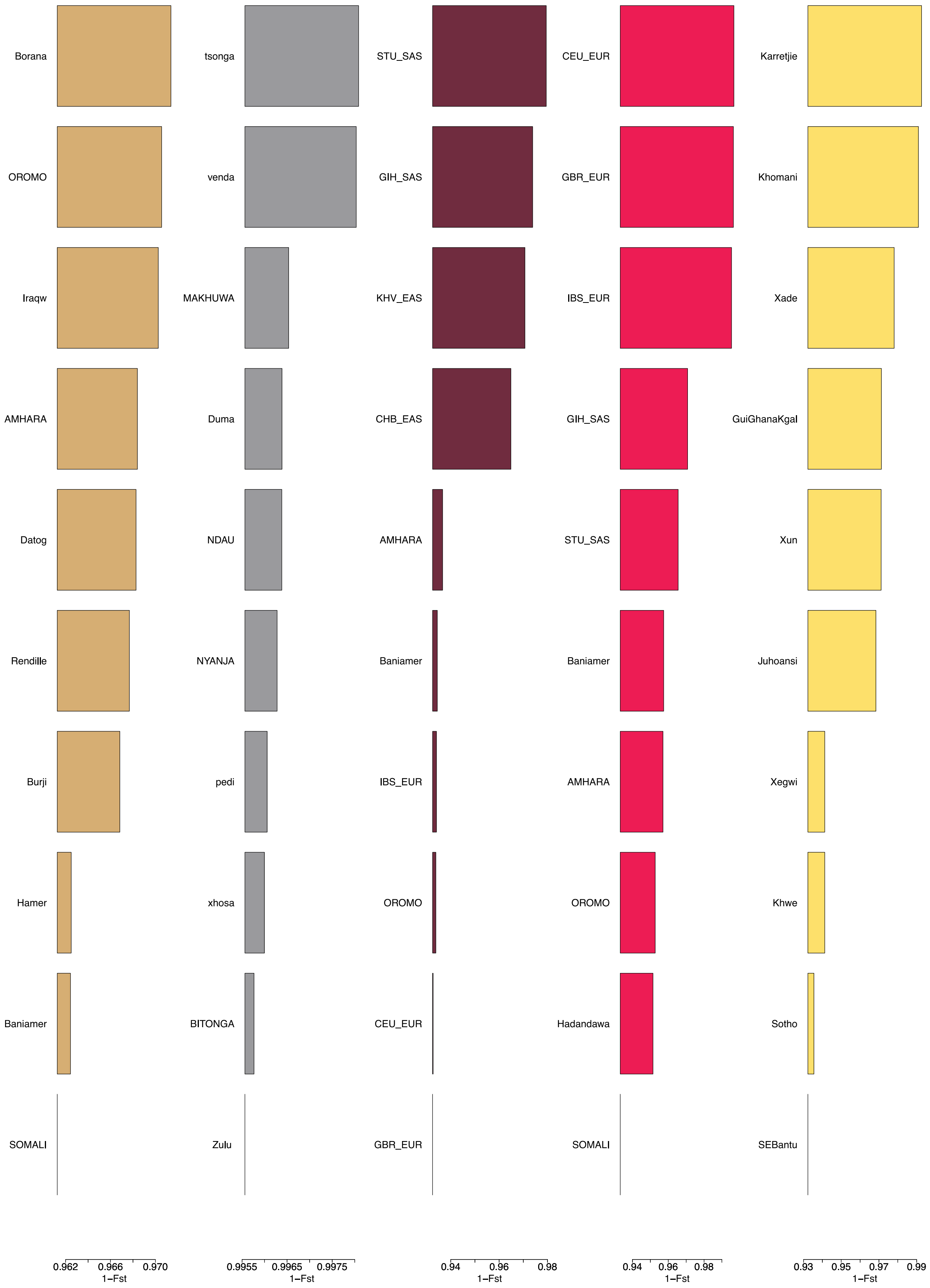

Figure S 21 – 1–F_{ST} estimates between local ancestral groups of the Heidelberg Hessequa-descendants and each donor panel. The values were obtained conditioned on a 5-way admixture model using MOSAIC. To each ancestral group is reported the top 10 "1–F_{ST}" values across the populations present in the dataset (Table S8).

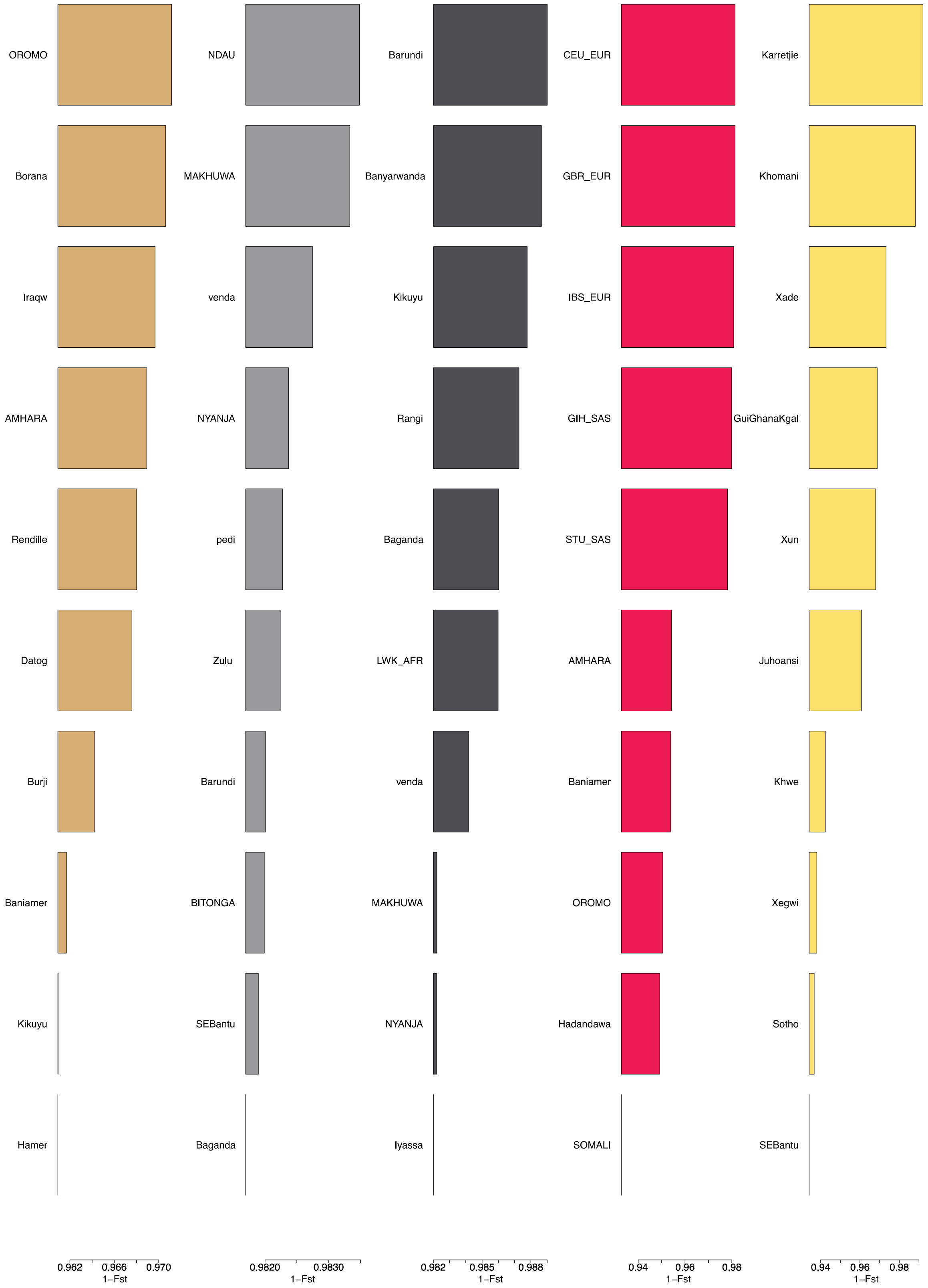

Figure S 22 – 1–F_{ST} estimates between local ancestral groups of the Melkhoutfontein Hessequa-descendants and each donor panel. The values were obtained conditioned on a 5-way admixture model using MOSAIC. To each ancestral group is reported the top 10 "1–F_{ST}" values across the populations present in the dataset (Table S8).

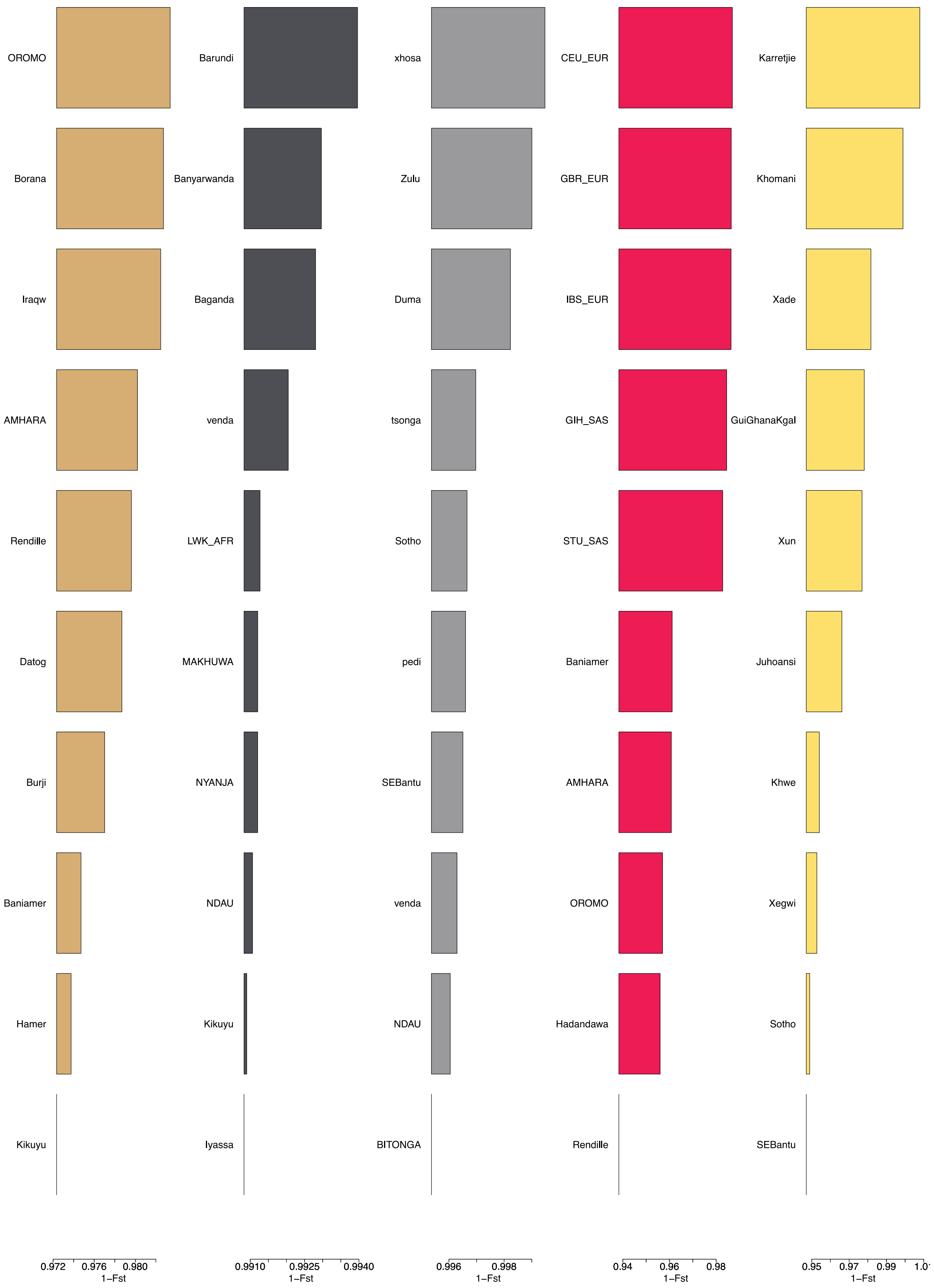

Figure S 23– 1–F_{ST} estimates between local ancestral groups of the Railton Hessequa-descendants and each donor panel. The values were obtained conditioned on a 5-way admixture model using MOSAIC. To each ancestral group is reported the top 10 "1–F_{ST}" values across the populations present in the dataset (Table S8).

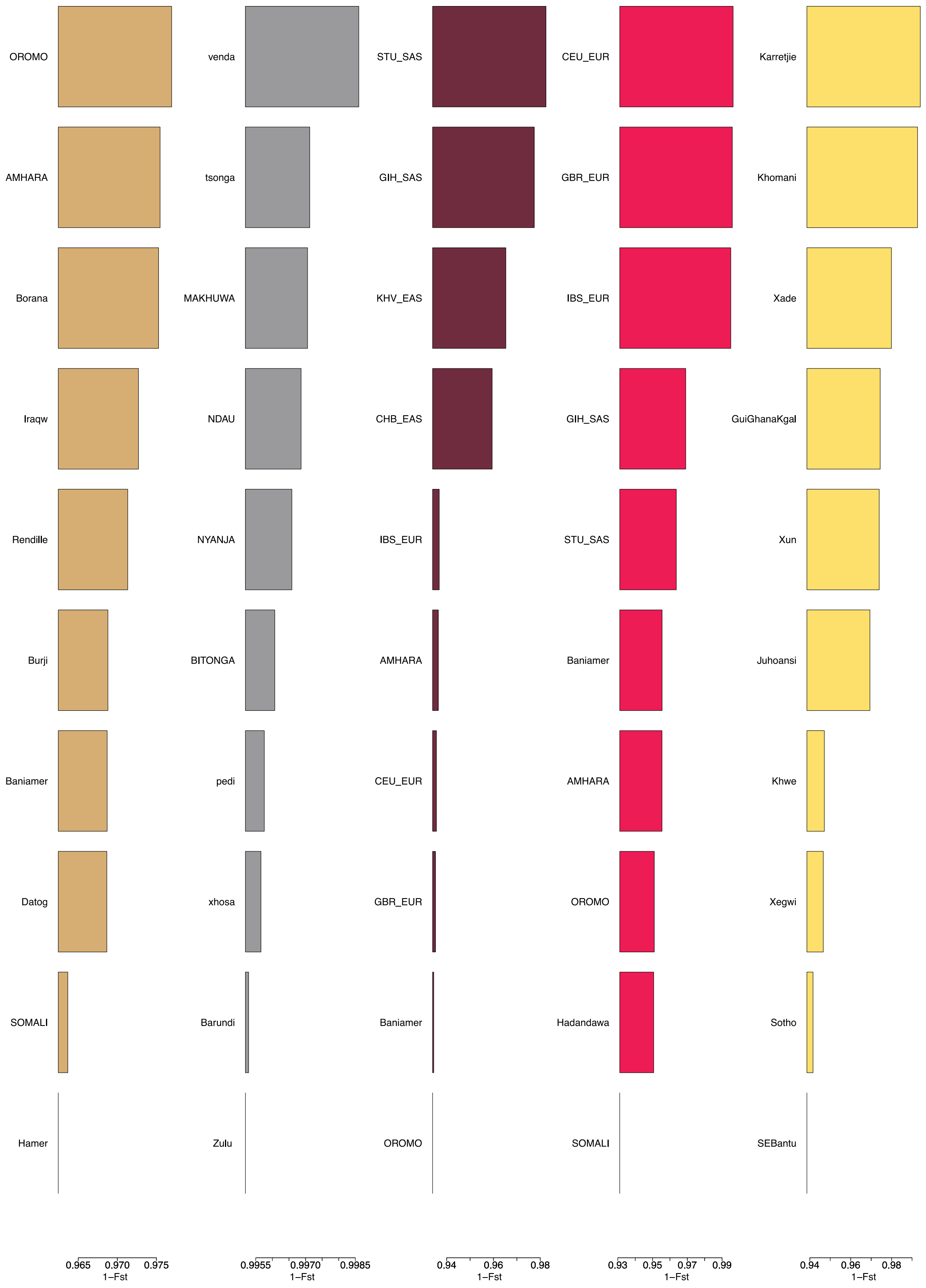

Figure S 24 – 1–F_{ST} estimates between local ancestral groups of the Riversdale Hessequa-descendants and each donor panel. The values were obtained conditioned on a 5-way admixture model using MOSAIC. To each ancestral group is reported the top 10 "1–F_{ST}" values across the populations present in the dataset (Table S8).

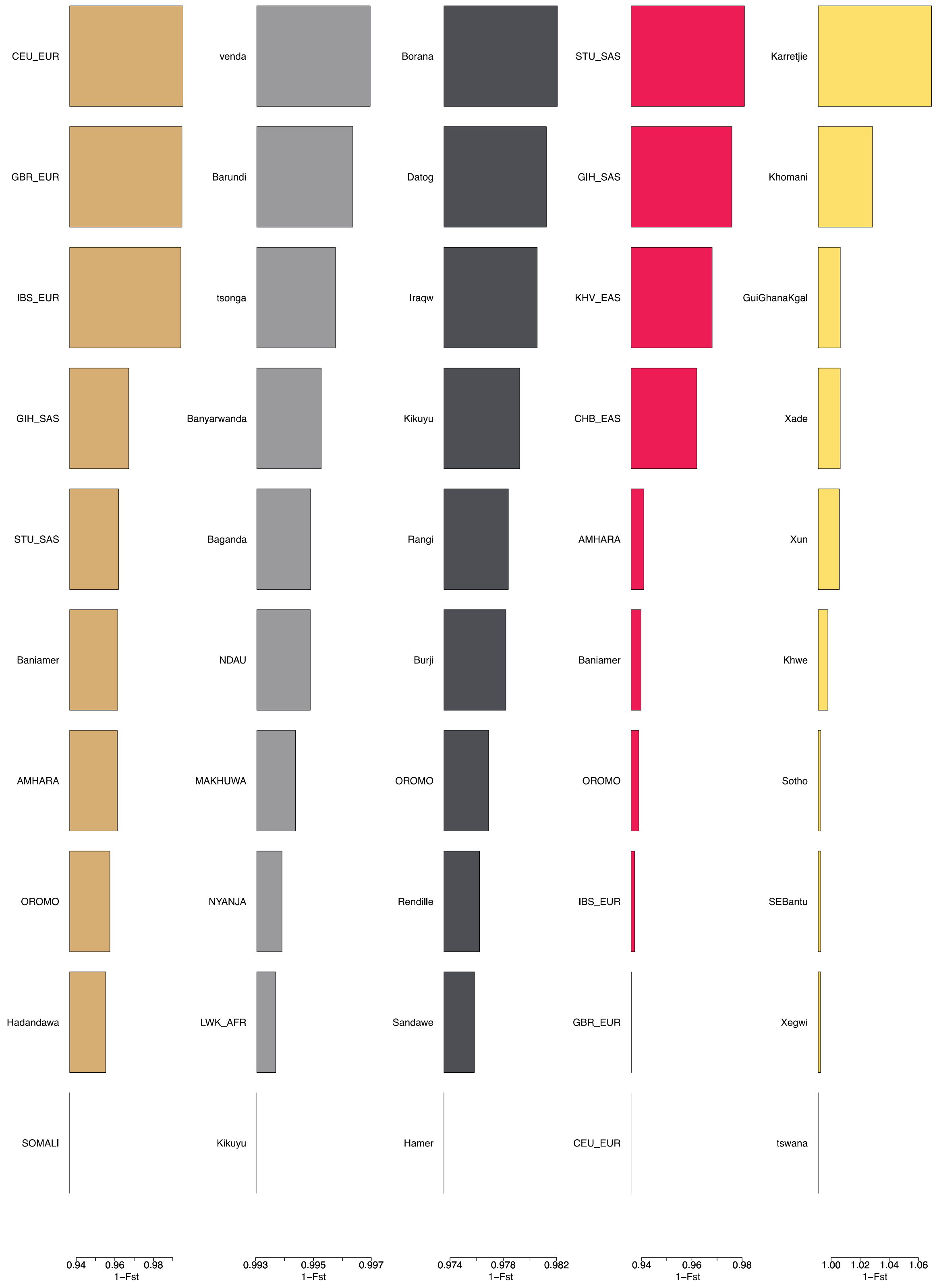

Figure S 25– 1–F_{ST} estimates between local ancestral groups of the Rotterdam Farm Hessequa-descendants and each donor panel. The values were obtained conditioned on a 5-way admixture model using MOSAIC. To each ancestral group is reported the top 10 "1–F_{ST}" values across the populations present in the dataset (Table S8).

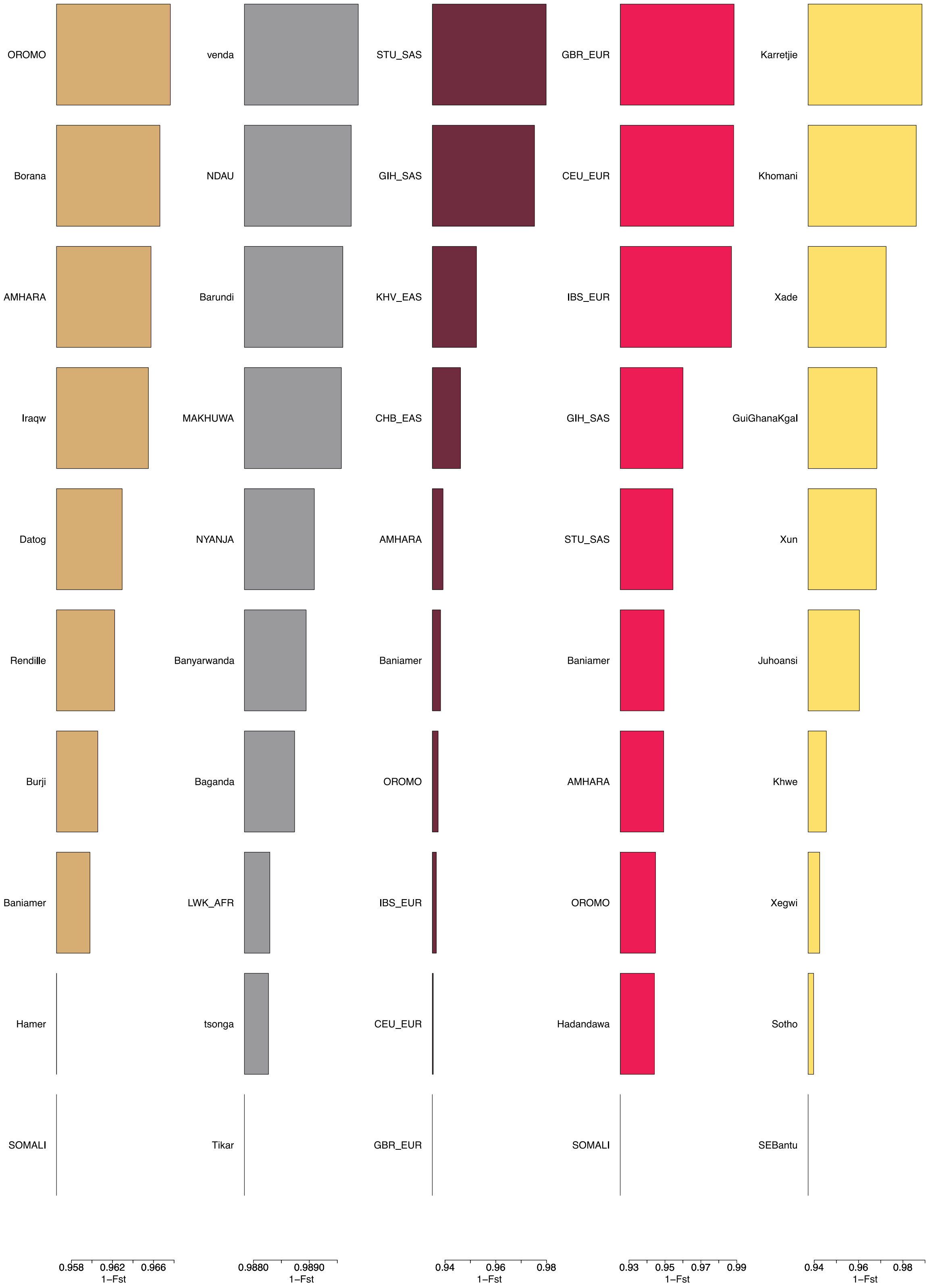

Figure S 26 – 1–F_{ST} estimates between local ancestral groups of the Slangriver Hessequa-descendants and each donor panel. The values were obtained conditioned on a 5-way admixture model using MOSAIC. To each ancestral group is reported the top 10 "1–F_{ST}" values across the populations present in the dataset (Table S8).

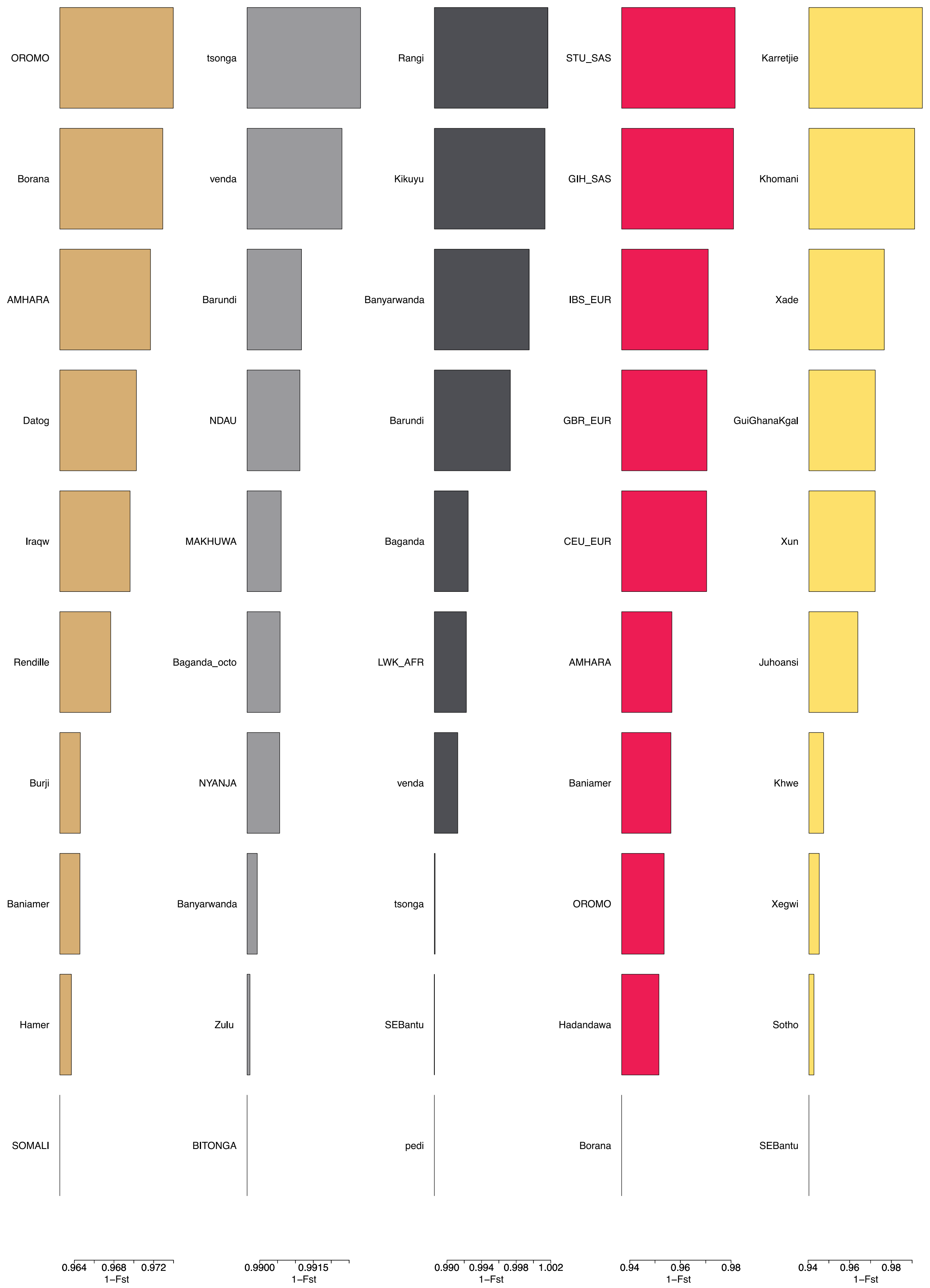

Figure S 27 – 1–F_{ST} estimates between local ancestral groups of the Stormsvlei Hessequa-descendants and each donor panel. The values were obtained conditioned on a 5-way admixture model using MOSAIC. To each ancestral group is reported the top 10 "1–F_{ST}" values across the populations present in the dataset (Table S8).

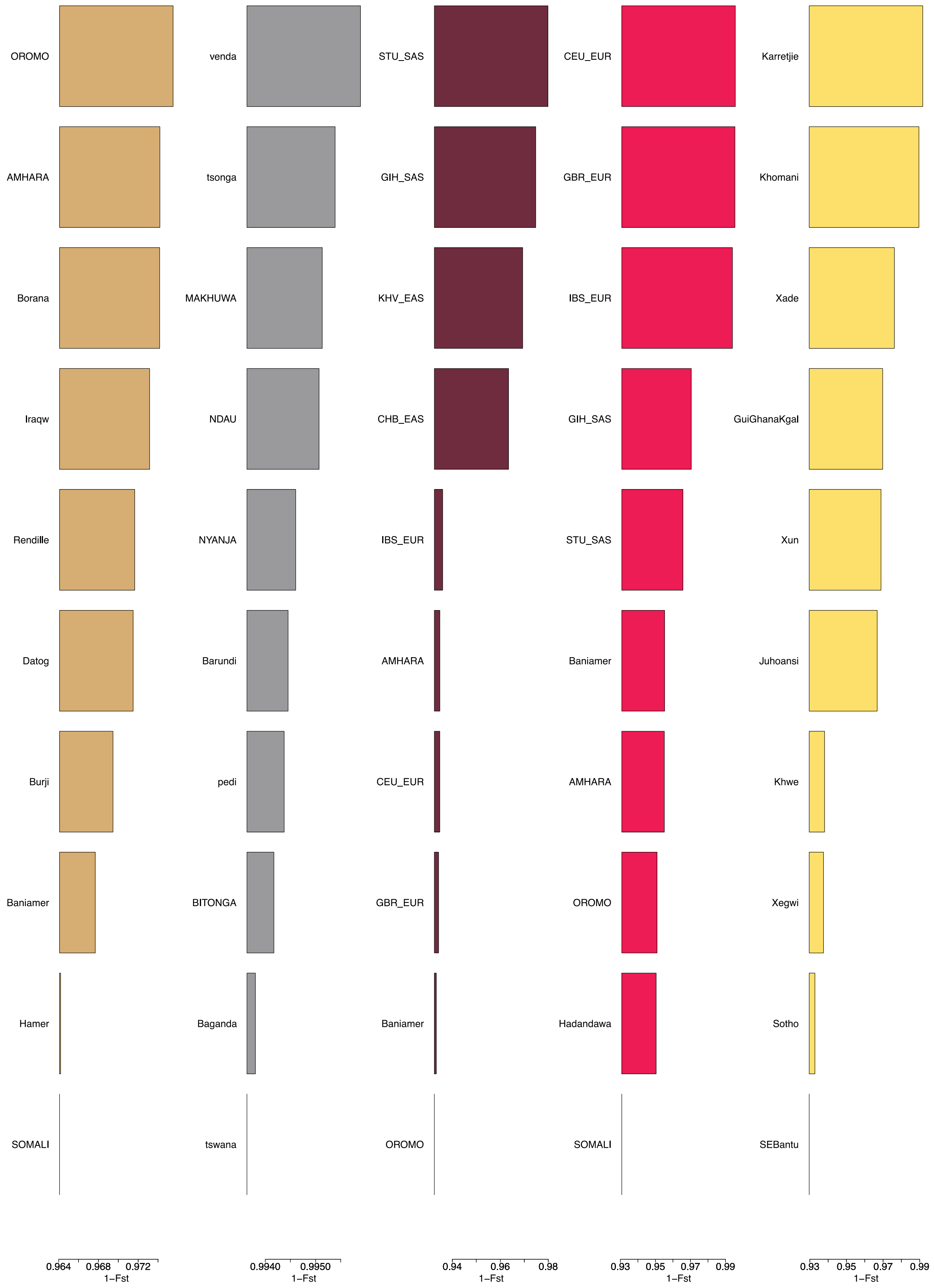

Figure S 28 – 1–F_{ST} estimates between local ancestral groups of the Suurbrack Hessequa-descendants and each donor panel. The values were obtained conditioned on a 5-way admixture model using MOSAIC. To each ancestral group is reported the top 10 "1–F_{ST}" values across the populations present in the dataset (Table S8).

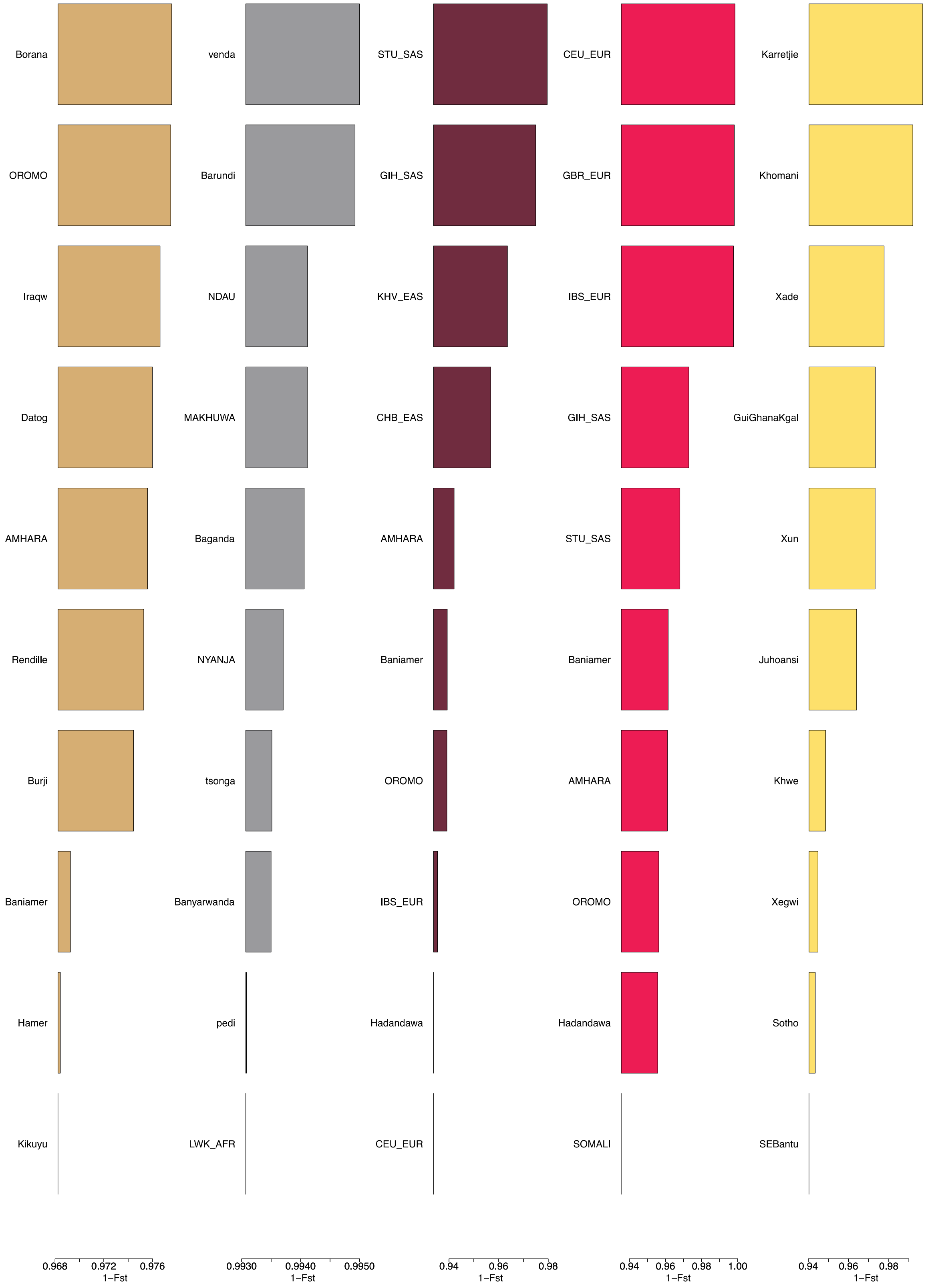

Figure S 29 – 1–F_{ST} estimates between local ancestral groups of the Swellendam Hessequa-descendants and each donor panel. The values were obtained conditioned on a 5-way admixture model using MOSAIC. To each ancestral group is reported the top 10 "1–F_{ST}" values across the populations present in the dataset (Table S8).

A

B

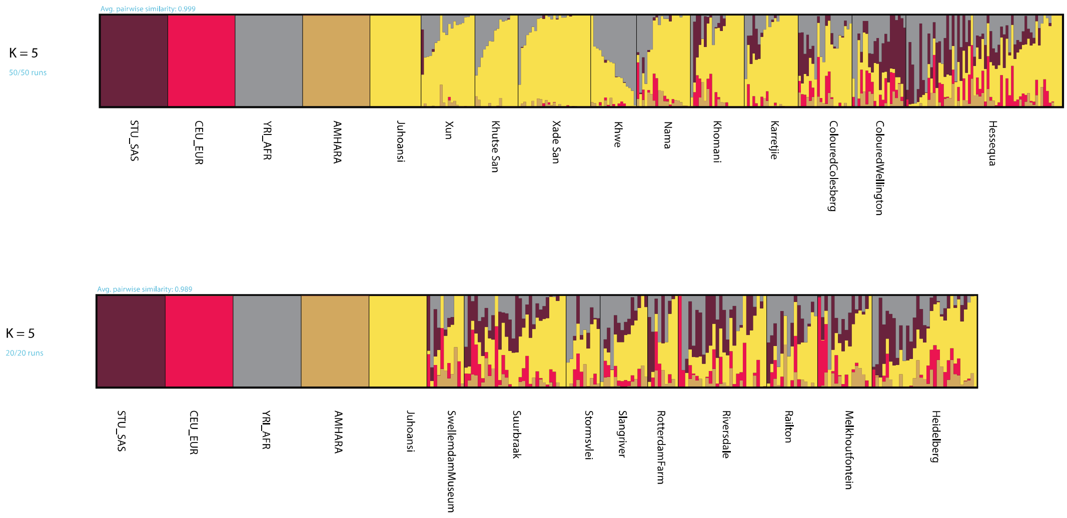

Figure S 30 – Supervised ADMIXTURE clustering analysis for the X-chromosome at K=5. A – Analysis performed among Khoe-San populations with an average East Africa ancestry higher than 2% in the autosomes and X-chromosome (Hessequa-descendants, Nama from Windhoek, Khwe, ǂKhomani and Coloured population of Wellington). B – Analysis performed in across all nine Hessequa-descendant sampling sites without filtering. The number of iterative runs that support the cluster assignment are given on the left of the figure.

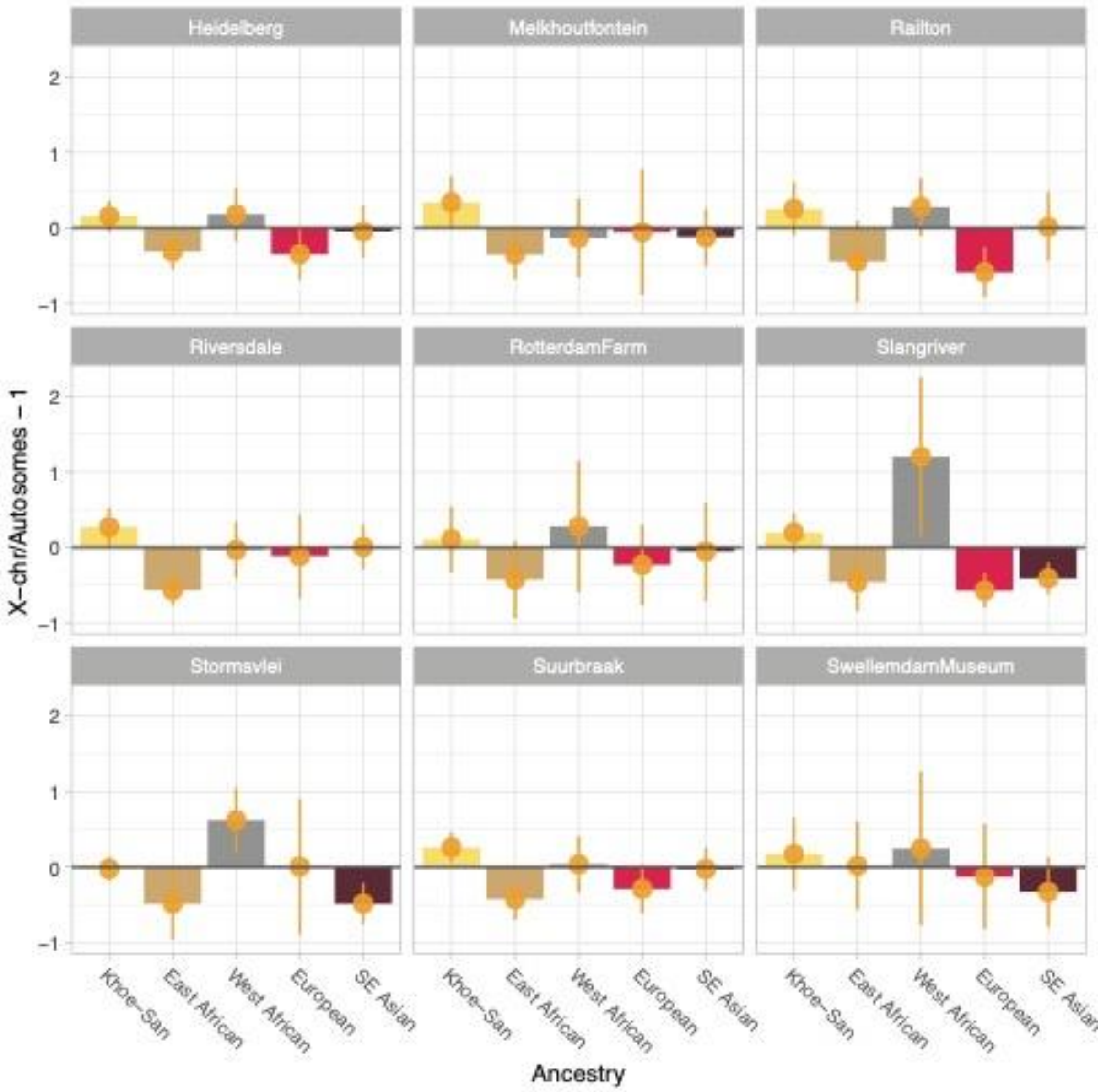

Figure S 31 – X-chromosome to autosomal ratio for each Hessequa-descendants sampling site. The ratio is based on the average ancestry proportion. Autosomal data is represented by the first 180 cM of chromosomes 1-6 and 7, 10 and 12. Error bars represent two standard deviations based on 100 random sampling bootstraps.
